# Colorectal cancer development is affected by the ECM molecule EMILIN-2 hinging on macrophage polarization via the TLR-4/MyD88 pathway

**DOI:** 10.1186/s13046-022-02271-y

**Published:** 2022-02-11

**Authors:** Eva Andreuzzi, Albina Fejza, Maurizio Polano, Evelina Poletto, Lucrezia Camicia, Greta Carobolante, Giulia Tarticchio, Federico Todaro, Emma Di Carlo, Melania Scarpa, Marco Scarpa, Alice Paulitti, Alessandra Capuano, Vincenzo Canzonieri, Stefania Maiero, Mara Fornasarig, Renato Cannizzaro, Roberto Doliana, Alfonso Colombatti, Paola Spessotto, Maurizio Mongiat

**Affiliations:** 1 Department of Research and Diagnosis, Division of Molecular Oncology, Centro di Riferimento Oncologico di Aviano (CRO) IRCCS, Aviano, Italy; 2 Experimental and Clinical Pharmacology Unit, Centro di Riferimento Oncologico di Aviano (CRO) IRCCS, Aviano, Italy; 3 Department of Medicine and Sciences of Aging, “G. d’Annunzio” University of Chieti-Pescara, Chieti, Italy; 4 Anatomic Pathology and Immuno-Oncology Unit, Center for Advanced Studies and Technology (CAST), “G. d’Annunzio” University of Chieti-Pescara, Chieti, Italy; 5 Ricerca Traslazionale Avanzata, Istituto Oncologico Veneto IOV – IRCCS, Padua, Italy; 6 Clinica Chirurgica I- Azienda Ospedaliera di Padova, Padua, Italy; 7 Pathology, Centro di Riferimento Oncologico di Aviano (CRO) IRCCS, Aviano, Italy; 8 Division of Oncological Gastroenterology, Centro di Riferimento Oncologico di Aviano (CRO) IRCCS, Aviano, Italy

**Keywords:** Extracellular matrix, Tumor microenvironment, Colorectal cancer

## Abstract

**Background:**

Colorectal cancer is one of the most frequent and deadly tumors. Among the key regulators of CRC growth and progression, the microenvironment has emerged as a crucial player and as a possible route for the development of new therapeutic opportunities. More specifically, the extracellular matrix acts directly on cancer cells and indirectly affecting the behavior of stromal and inflammatory cells, as well as the bioavailability of growth factors. Among the ECM molecules, EMILIN-2 is frequently down-regulated by methylation in CRC and the purpose of this study was to verify the impact of EMILIN-2 loss in CRC development and its possible value as a prognostic biomarker.

**Methods:**

The AOM/DSS CRC protocol was applied to *Emilin-2* null and *wild type* mice. Tumor development was monitored by endoscopy, the molecular analyses performed by IHC, IF and WB and the immune subpopulations characterized by flow cytometry. Ex vivo cultures of monocyte/macrophages from the murine models were used to verify the molecular pathways. Publicly available datasets were exploited to determine the CRC patients’ expression profile; Spearman’s correlation analyses and Cox regression were applied to evaluate the association with the inflammatory response; the clinical outcome was predicted by Kaplan-Meier survival curves. Pearson correlation analyses were also applied to a cohort of patients enrolled in our Institute.

**Results:**

In preclinical settings, loss of EMILIN-2 associated with an increased number of tumor lesions upon AOM/DSS treatment. In addition, in the early stages of the disease, the *Emilin-2* knockout mice displayed a myeloid-derived suppressor cells-rich infiltrate. Instead, in the late stages, lack of EMILIN-2 associated with a decreased number of M1 macrophages, resulting in a higher percentage of the tumor-promoting M2 macrophages. Mechanistically, EMILIN-2 triggered the activation of the Toll-like Receptor 4/MyD88/NF-κB pathway, instrumental for the polarization of macrophages towards the M1 phenotype. Accordingly, dataset and immunofluorescence analyses indicated that low EMILIN-2 expression levels correlated with an increased M2/M1 ratio and with poor CRC patients’ prognosis.

**Conclusions:**

These novel results indicate that EMILIN-2 is a key regulator of the tumor-associated inflammatory environment and may represent a promising prognostic biomarker for CRC patients.

**Supplementary Information:**

The online version contains supplementary material available at 10.1186/s13046-022-02271-y.

## Background

Colorectal cancer (CRC) is the third most common malignancy and one of the major causes of cancer-related deaths [1]. Resection of both primary and metastatic lesions ensures the best prognosis for these patients, however post-intervention recurrence is very common due to disseminated latent or therapy-resistant tumor cells [2]. Accumulating evidence suggest that tumor progression and recurrence are governed not only by genetic changes intrinsic to cancer cells, but also by microenvironmental cues [3]. The role of the immune system in modulating CRC growth and progression has been extensively demonstrated [4]. Inflammatory responses play a decisive role in different stages of tumor development, including initiation, promotion, malignant conversion, invasion and metastasis [5]. Recent observations highlighted that the immune landscape of CRC impacts also on the efficacy of conventional chemotherapies and of targeted therapies and affects the onset of possible mechanisms of resistance [6–11].

It is currently accepted that the immune system plays a dual role in cancer. On one side, it can suppress tumor growth by destroying cancer cells or inhibiting their outgrowth, a process that is known as cancer immunosurveillance [12], chiefly mediated by myeloid derived suppressor cells (MDSC) [13]. On the other side, it can also promote tumor progression, either by selecting for tumor cells that are more adaptable to survive in an immunocompetent host (immunoselection), or by establishing conditions within the tumor microenvironment that facilitate tumor growth (immunosubversion) [4]. All these mechanisms are referred to as tumor immunoediting [14]. One of the main tumor-infiltrating immune cell types involved in immunoediting are tumor-associated macrophages. Two functionally contrasting macrophage subtypes have been described, namely classical activated M1 macrophages and alternatively activated tumor-promoting M2 macrophages. Interestingly, a high degree of plasticity characterizes these two cell subtypes, which can easily shift from one phenotype to the other, despite the microenvironmental changes responsible for this phenomenon have not been completely uncovered [15].

One vital microenvironmental component is the extracellular matrix (ECM) since it plays both direct and indirect roles in modulating tumor cell behavior. In the tumor microenvironment, the ECM exerts pleiotropic effects regulating cell adhesion and migration, controlling tumor infiltration by macrophages or other leukocytes, affecting tumor angiogenesis and regulating growth factor availability, as well as receptor signaling [16–24]. EMILIN-2 is an ECM molecule belonging to the EDEN protein family [25, 26] and exerts a suppressive function in a number of tumor types [27, 28]. In the tumor microenvironment, EMILIN-2 affects the activation of the Wnt/β-catenin signaling pathway [29], a key regulator of CRC development, thus down-modulating cell proliferation and migration. Like other members of this family such as Multimerin-2 [30–32], EMILIN-2 also influences angiogenesis [27, 33, 34], triggering IL-8 expression via the EGFR/EGF pathway, thus affecting vessel development and perfusion [35]. EMILIN-2 expression is often down-regulated during tumor progression [36, 37], however the impact of this loss has not been explored thus far. We verified that the expression of EMILIN-2 was variable among the CRC patients, thus we sought to evaluate the effects of EMILIN-2 loss in CRC development, also considering its role in modulating the Wnt/β-catenin signaling pathway [29]. To this end, we took advantage of the *Emilin-2* null mouse model. Contrary to our expectations, we found that loss of EMILIN-2 did not affect the activation of the Wnt/β-catenin signaling pathway, instead we unveiled a previously undescribed immunomodulatory function for this molecule. In fact, low EMILIN-2 levels correlated with a low M1/M2 macrophage ratio infiltrating the tumor tissue, and with poor CRC patient prognosis. Based on these results we envision that EMILIN-2 may represent a valid prognostic marker for CRC patients.

## Methods

### Cell cultures

The 293-EBNA, 293FT, U937, THP-1, HTC-116, HT-29 cell lines were obtained from ATCC (Manassas, VA, USA). NHDF (normal human dermal fibroblasts) cells were obtained from LONZA (Basel, Switzerland). U937, THP-1, HTC-116, HT-29 cells were cultured in RPMI medium (LONZA, Basel, Switzerland) with 10% fetal bovine serum; 293-EBNA in Dulbecco’s modified Eagle medium (LONZA, Basel, Switzerland) with 10% fetal bovine serum containing 250 μg/ml G418; NHDF and 293FT cells were cultured in Dulbecco’s modified Eagle medium (LONZA, Basel, Switzerland) with 10% fetal bovine serum. All the cells were maintained at 37 ̊C under a humidified atmosphere containing 5% CO_2_ and verified to be free of mycoplasma contamination using the MycoAlert™ Mycoplasma Detection kit (LT07–318, LONZA).

### Patients

For this study, 23 patients with colorectal cancer were consecutively enrolled. Patients were examined before chemotherapy or surgical intervention. The bioptic samples were obtained with macrobiopy (COOK Medical, Ireland) at the end of examination. The clinical parameters are reported in Table S1.

### RT-qPCR, cell transfection and recombinant protein production

Total RNA was isolated from homogenized tissues or cell lines with Trizol and reverse transcribed using AMV-RT (Promega, Milan, Italy). Semi-quantitative endpoint reactions were performed with GoTaq DNA polymerase (Promega, Milan, Italy) and Real-time PCRs with iQ™ SYBR® Green Supermix (Bio-Rad, Milan, Italy). The oligonucleotide sequences are listed in Table S2.

Cell transfection was carried out using the FuGene6 reagent (Promega, Milan, Italy). For recombinant EMILIN-2 production, E293 cells were transfected with the pCEP-Pu-EMILIN-2 or the empty vector and were then selected with 250 μg/ml G418 and 0.5 μg/ml puromycin. Confluent cells were then incubated in serum-free medium for 48 h and the conditioned medium collected; the His-tagged protein was then purified with Ni-NTA beads (Qiagen GmbH, Hilden, Germany).

### Western blot analysis

Cells and mouse CRC tumors were lysed in cold RIPA buffer (150 mM NaCl, 10 mM Tris, 0.1% SDS, 1% Triton X-100, 1% sodium deoxycholate, 5 mM EDTA) containing a protease inhibitor cocktail (Roche Diagnostics S.p.a., Milan, Italy). Proteins resolved in 4 to 20% Criterion Precast Gels (Bio-Rad, Milan, Italy) were transferred onto Hybond-ECL nitrocellulose membranes, blocked with 5% dry milk in TBS-T buffer, probed with the appropriate antibodies: the anti-EMILIN-2 antibodies were obtained as described [27, 28]; anti-β-actin (cat.4967), anti-pSTAT3 (cat. 9145), anti-pNF-κB (cat. 3033) were from Cell Signaling (Danvers, MA, USA); anti-vinculine (cat.sc-7649), anti-TLR4 (cat. sc-293,072), anti-STAT3 (c20, cat. sc-482), anti-pSTAT1 (cat. sc-8394), anti-STAT1 (cat. sc-417), anti-NF-κB (cat. sc-372), anti-MyD88 (cat. sc-74,532) from Santa Cruz (Dallas, USA). The filters were developed using enhanced chemiluminescence (Amersham, Milan, Italy) or the Odyssey Infrared Imaging System (Li-COR Biosciences, Lincoln, USA).

### Histopathology, immunohistochemistry and immunofluorescence

The murine colon samples were sectioned from the ileocecal valve to the anus, washed in ice-cold PBS, fixed in 10% neutral buffered formalin, embedded in paraffin, and the 4 μm sections stained with hematoxylin and eosin (H&E). IHC was performed on paraffin-embedded sections as described [38] using the indicated antibodies. GR-1 and Mac-1 positive cells were counted in 8 randomly chosen fields under a microscope (magnification: 40x objective and 10x ocular lens; 0.180 mm^2^ per field). For the immunofluorescence analyses in human samples, 7 μm frozen colon samples were obtained and incubated with the primary antibodies overnight at 4 °C, the specific AlexaFluor secondary antibodies were applied for 1 h at RT. Nuclei were visualized with TO-PRO™-3. The following antibodies were used: anti-GR1 (cat. 108,401; clone RB6–8C5) from BioLegend (San Diego, CA); anti-Mac-1 (cat. ab259372), anti-CD163 (cat. Ab182422), anti HLA-DR (cat. Ab92511), anti-IL4R (cat. Ab203398) and anti-CD86 (cat. Ab239075) from Abcam (Cambridge, UK). TO-PRO™-3 (T3605) was from Invitrogen (Milan, Italy). Secondary AlexaFluor-conjugated antibodies were from Invitrogen (Milan, Italy), secondary HRP antibodies from Amersham (Milan, Italy).

### MTT and TUNEL assay

10^4^ HT-29 or HCT116 cells were plated in 96 well plate, let adhere and incubated with 5 μg/ml EMILIN-2 or PBS for 24, 48 and 72 h. Cells were incubated for 3 h with 5 mg/ml MTT and absorbance detected at 560 nm. The results were reported as % of cell proliferation. The apoptotic rate was determined using the Cell Death Detection ELISA PLUS TUNEL assay (Roche Diagnostics S.p.a., Milan, Italy).

### Soft agar colony assays

HCT-116 and HT-29 cells, 5 × 10^3^ cells/well, were included in 0.4% low melting-point agarose, placed on top of a 0.6% agarose layer, challenged with EMILIN-2 (5 μg/ml) or PBS and pictures were taken after 10 days. The colony number and area of clones formed by more than 10 cells were measured using Image Tool software.

### Migration assay

To evaluate cell migration, Transwell® chambers (Corning, Inc., New York, NY, USA) were used. 20 × 10^3^ HCT-116 and HT-29 cells were seeded into the upper chamber in serum free RPMI containing EMILIN-2 (5 μg/ml) or PBS; serum free RPMI was added to the lower chamber. The cells were allowed to migrate for 6 h at 37 °C in 5% CO_2_. The migrated cells were fixed with 4% paraformaldehyde and stained with crystal violet (0.5%) and counted.

### U937 and THP-1 activation, proliferation and Annexin V staining

U937 were activated with TPA (50 ng/ml) (12-O-tetradecanoylphorbol-13-acetate from Sigma-Aldrich, Milan, Italy) for 72 h w/wo recombinant EMILIN-2 (5 μg/ml) and CD11b expression was evaluated by flow cytometry. 2 × 10^5^ cells/ml were seeded and incubated with 5 μg/ml EMILIN-2 or PBS for 24, 48 and 72 h, and cell proliferation was assessed by counting. To evaluate the percentage of apoptotic cells, 2 × 10^5^ cells/ml were treated with EMILIN-2 (5 μg/ml) or PBS for 24, 48 and 72 h and stained for 15 min with the FITC Annexin V antibody and with propidium iodide (PI) (cat. 556,420 and cat. 556,463, respectively, BD Bioscences, Milano, Italy) and analyzed by flow cytometry (BD FACSCanto II flow cytometer) and the BD FACSDiva v8.0.1 software (BD Biosciences, Milano, Italy). Cells were treated with 500 μM H_2_O_2_ for 1 h as positive control.

### Adhesion assay

Naive and TPA activated U937 cells (50 ng/ml for 72 h) were kept in serum free RPMI medium overnight. Wells were pre-coated overnight with 5 μg/ml EMILIN-2 or Fibronectin or PBS, followed by incubation in blocking buffer (2% BSA in PBS) for 1 h. Cells were let adhere for 4 h. Unattached cells were washed away with PBS and adhered cells were stained with crystal violet and counted.

### Co-immunoprecipitation

THP-1 cells were activated with TPA (50 ng/ml), treated with recombinant EMILIN-2 (2 μg/ml), LPS (1 mg/ml) or PBS. After stimulation, whole-cell extracts were prepared with HNTG lysis buffer and incubated overnight with the anti-MyD88 antibody, together with Protein A/G beads (Santa Cruz). Beads were then washed with PBS and immunoprecipitates were eluted with sodium dodecyl sulfate (SDS) loading buffer, resolved in 4 to 20% Criterion Precast Gels (Bio-Rad, Milan, Italy), transferred onto Hybond-ECL nitrocellulose membranes and further incubated with the appropriate antibodies.

### Polarization assay

THP-1 cells were first activated with TPA (50 ng/ml) (12-O-tetradecanoylphorbol-13-acetate from Sigma-Aldrich, Milan, Italy), and after 24 h placed in fresh complete RPMI for additional 24 h. Next, the cells were challenged for 24 h with the specific polarization stimuli: LPS (250 ng/ml) and IFN-γ (20 ng/ml) for M1 and IL-4 (40 ng/ml) for M2, in the presence of recombinant EMILIN-2 (2 μg/ml) or PBS and in combination with the TAK-242 (1 μM) and JSH-23 (10 μM) inhibitors. After 24 h cell lysate and RNA were obtained as previously described.

### Knockdown of TLR-4

For lentiviral production, 293FT cells were co-transfected, using a standard calcium phosphate precipitation, with the lentiviral based shRNA constructs (pLKO) and lentiviral vectors pPAX2 and pVSV-G (Addgene, Watertown, USA). 48 h after transfection, medium containing viral particles was collected, supplemented with 8 mg/ml polybrene (Sigma-Aldrich, Milan, It) and used to transduce TPA-activated THP-1 cells. After 48 h the cells were treated with recombinant EMILIN-2 (2 μg/ml) or PBS. After 24 h cell lysate and RNA were collected. The knockdown efficiency was confirmed by Western Blot. pLKO for control (SHC002) and the specific TLR-4 shRNAs (shTLR-4 #1: TRCN0000056895 and shTLR-4 #2: TRCN0000056893) were purchased from Sigma-Aldrich (Milan, Itay).

### In vivo tumor growth

*Wild type* (*wt*) and *Emilin-2*^−/−^ C57BL/6 J 5 weeks-old mice were treated with AOM/DSS, as previously described [39]. Briefly, animals were injected intraperitoneally with azoxymethane (AOM,cat. A5486, Sigma-Aldrich, Milan, Italy) (12.5 mg/kg) and after 7 days 2% Dextran Sodium Sulfate (DSS, MW 36,000–50,000 Da, cat. 9011-18-1, MP Biomedicals, USA) was added to the drinking water for 7 days. For sporadic CRC, mice were injected with AOM (12.5 mg/kg) once a week for 6 weeks. For chronic inflammation, DSS was administered for 7 days followed by 10 days of fresh water and the cycle repeated 7 times.

### Isolation of bone marrow-derived macrophages (BMmacs)

BMmacs were isolated and differentiated from murine tibias and femurs as previously described [40]. Red blood cells were lysed in ammonium chloride potassium buffer for 4 min and 2 × 10^6^ cells/ml were differentiated with 30 ng/ml recombinant M-CSF for 7 days. Following differentiation, the medium was supplemented with IFN-γ and TNF-α (for M1 polarization) or with IL-4 and IL-10 (for M2 polarization) in the presence of recombinant EMILIN-2 (2 μg/ml) or PBS. After 24 h mRNA was harvested with Trizol and processed for expression analyses.

### Flow cytometry analyses

Single cell suspension of colon cells were isolated as previously described [41, 42] and analyzed by flow cytometry. Cells were stained with the following antibody panels: panel 1) CD45, CD11b, CD11c, F4/80, MHC II, Ly-6C, and Ly-6G; panel 2) CD45, CD11b, CD3, CD4, CD8, CD19, and MHC II. Flow cytometry was performed with the FACS LSR Fortessa (BD Biosciences) and data were analyzed using DIVA software (BD Biosciences). The following antibodies were used: anti-F4/80 BV421 (cat. 565,411), anti-Ly-6C Alexa Fluor 700 (cat. 561,237), anti-CD11c PE (cat. 561,044), anti-MHC II BV650 (cat. 563,415), anti-CD3 BV510 (cat. 563,024) from BD Bioscences (Milano, Italy); anti-CD11b PerCP-Cy5.5 (cat. 45–0112), anti-CD4 Alexa Fluor 700 (cat. 56–0041), anti-Ly-6G FITC (cat. 11–5931), anti-CD45 Pe-Cy5 (cat. 15–0451), anti-CD8 PE (cat. 12–0083), anti-CD19 FITC (cat. 11–0193) from EBiosciences (San Diego, CA, USA).

### Bioinformatics and statistical analysis

Transcriptomics data from Cancer Genome Atlas of Colorectal Cancer type were downloaded from GDC Data portal (https://portal.gdc.cancer.gov/ accessed on 12 March 2021) and proteomics data were downloaded from Vasaikar et al. using cBioportal (The cBio Cancer Genomics Portal: An Open Platform for Exploring Multidimensional Cancer Genomics Data, accessed on 12 March 2021) [43]. The *EMILIN-2* gene expression profiles were analyzed using a dataset of colon cancer patients from the GEO database (GSE35834) [44]. This dataset reports expression estimations, obtained with Affymetrix Human Genome U133 Plus 2.0 arrays. Cox regression and Kaplan-Meier survival curves were computed using R (version 3.6.1) with *survival* and *survminer* packages. Survival curves were compared with the log-rank test. Survival analyses were performed according to Liu et al. [45] from the TCGA-COAD cohort.

The composition and density of immune cells in the tumor microenvironment (TME) was assessed using the *immunedeconv* tool as described by Sturm et al. [46] and the *timer2* tool [47]. *EMILIN-2* methylation profile on colorectal cancer was obtained using MEXPRESS (http://mexpress.be) [48].

Statistical analyses were performed with the SigmaPlot and GraphPad Prism 6 software (Graphpad) and the values represent the mean ± standard deviation obtained with not less than three measurements on randomized samples. The statistical significance of the differences was determined by the two-sided Student’s t test for the comparisons between two groups; for more than two groups, the ANOVA 1-way analysis of variance was used, according to the Bonferroni method. For the in vivo experiments, mice were randomly assigned to treatment groups. Differences were considered statistically significant when *P* ≤ 0.05.

## Results

### EMILIN-2 levels are down-regulated in the CRC microenvironment

**Fig. 1:**
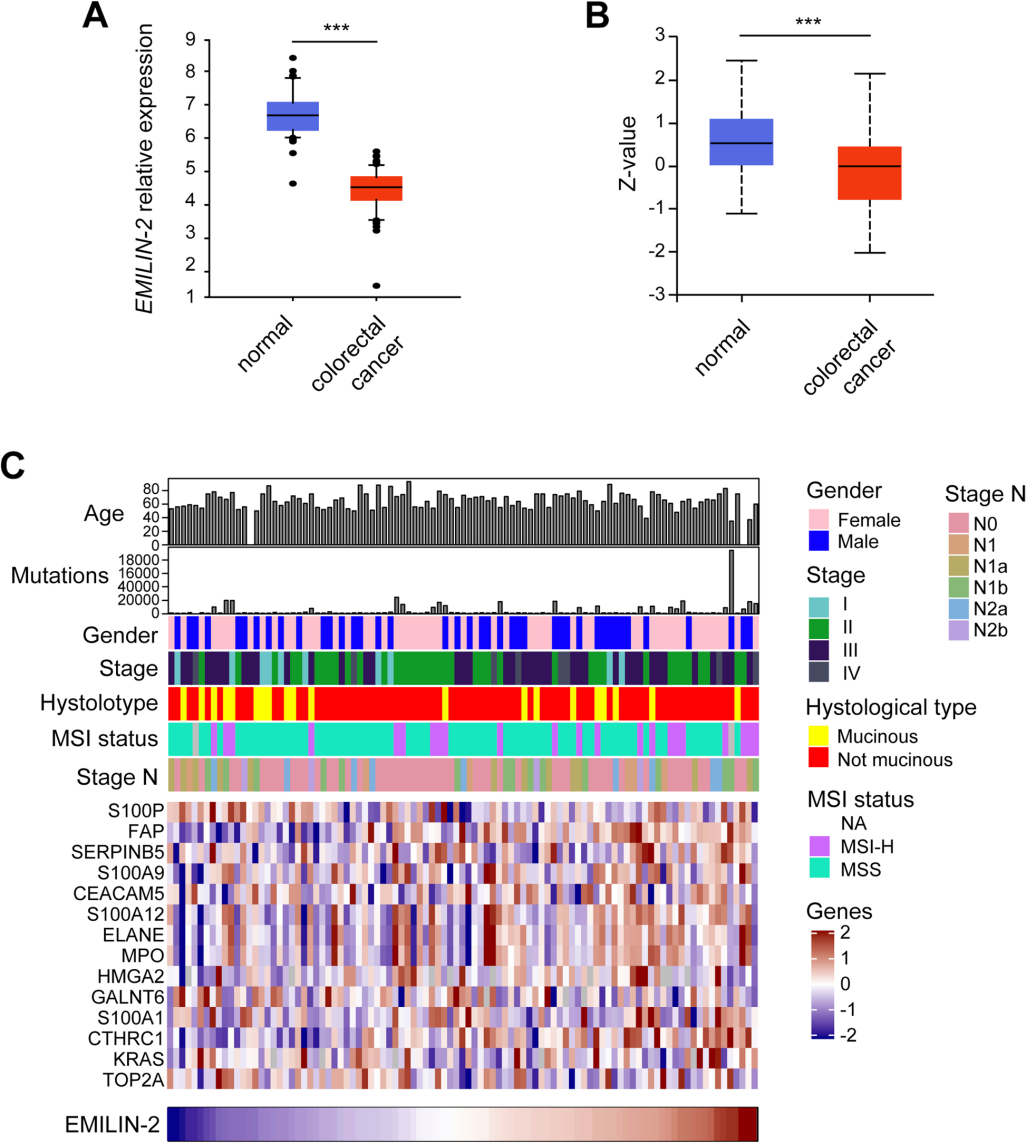
EMILIN-2 expression is down-regulated in CRC. **A** *EMILIN-2* gene expression levels in healthy (normal) and neoplastic (colorectal cancer) human samples according to the GEO public database (ID: GSE3629; healthy *n* = 53, neoplastic *n* = 68). **B** EMILIN-2 protein levels in healthy (normal; *n* = 100) and neoplastic (colorectal cancer; *n* = 97) human samples according to the cBioportal public dataset. **C** Evaluation of molecular and clinical parameters in relation to the levels of EMILIN-2 from the CRC patients’ cohort reported in B. Graphs in A and B represent the mean ± SD; *P* values were obtained using the paired Student’s t-test; ***P < 0.001

To outline the profile of EMILIN-2 in the context of CRC, we used different omic approaches. The epigenetic modulation of gene expression has a pivotal role in CRC development and is emerging as an important biomarker to guide the therapeutic choices [49]. Thus, based on the finding that epigenetic modifications of *EMILIN-2* gene occur in a number of solid tumors [36], we first evaluated the levels of methylation of the *EMILIN-2* gene in the TCGA COAD cohort of 477 CRC patients and found that the *EMILIN-2* gene carries many methylation sites (Fig. S1). Furthermore, the MEXPRESS analyses indicated that a higher methylation of the *EMILIN-2* gene was associated with higher rate of braf mutations (*p* = 0.040), higher number of colon polyps (*p* = 5.7e10^− 6^), increased lymphatic invasion (*p* = 0.040) as well as with the pathologic M status (*p* = 0.015) (Fig. S1). The down-regulation of *EMILIN-2* expression in CRC patients was also confirmed at the mRNA level, as assessed in a cohort of 121 patients (GEO ID: GSE3629 [44]), as well as at the protein level in a cohort of 110 patients available at the cBioportal public dataset [50] (Fig. 1 A,B, respectively). Despite overall reduced, we observed a variability of EMILIN-2 among the CRC samples analyzed (Fig. 1C and Fig. S2). The same dataset was interrogated to verify if the expression of EMILIN-2 associated with known CRC molecular and clinically relevant parameters. We found that the expression of EMILIN-2 did not correlate with the gender of the patients, the stage, histological type, MSI status or stage N of the disease. However, among the various markers taken into account we found that low EMILIN-2 expression associated with high levels of the calcium binding protein S100P (*P* = 0.0271), whose expression is linked to poor overall survival in CRC patients [51].

### *Emilin-2* null mice are more susceptible to AOM/DSS induced CRC

**Fig. 2:**
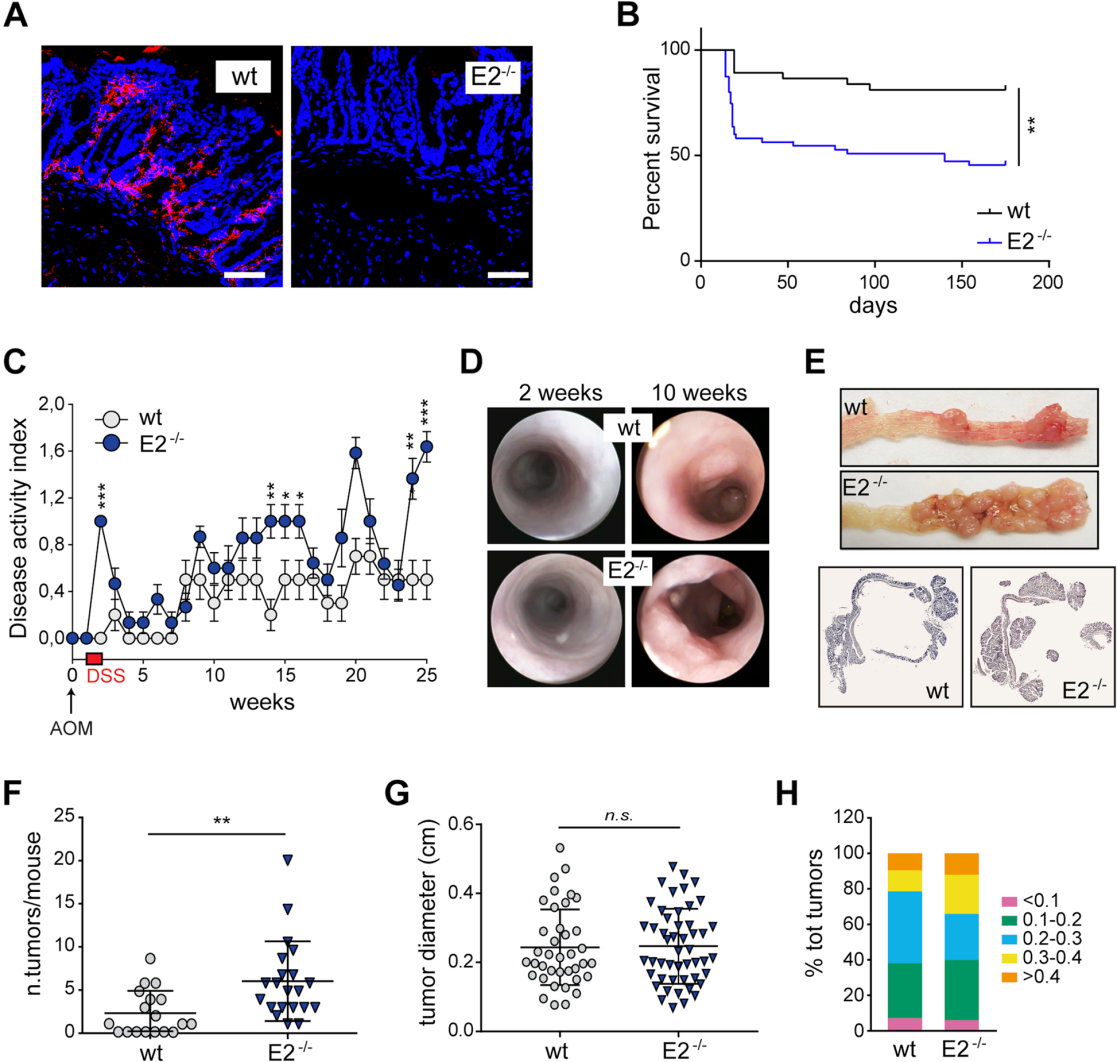
Ablation of EMILIN-2 associates with exacerbated tumorigenesis upon AOM/DSS treatment in mice. **A** EMILIN-2 staining in the colonic mucosa of *wild type* (wt) and *Emilin-2*^*−/−*^ (E2^−/−^) mice; Blue: nuclei; red: EMILIN-2; scale bar = 50 μm. **B** Kaplan-Meier graph showing the overall survival of *wild type* (wt; *n* = 30) and *Emilin-2*^*−/−*^ (E2^−/−^; *n* = 50) mice treated with AOM/DSS. Log-rank test, *P* = 0,0025. **C** Graph showing the Disease Activity Index (DAI) assessed in *wild type* (wt; *n* = 18) and *Emilin-2*^*−/−*^ (E2^−/−;^ *n* = 21) mice during AOM/DSS treatment. **D** Endoscopic images of the colonic mucosa of *wild type* (wt) and *Emilin-2*^*−/−*^ (E2^−/−^) mice at 2 and 10 weeks from the AOM administration. **E** Top, macroscopic images of the tumors developed in *wild type* (wt) and *Emilin-2*^*−/−*^ (E2^−/−^) mice 25 weeks after AOM administration; bottom, H&E staining of the colon sections from the experiment reported in **E**. **F** Graph indicating the number of tumors per mouse developed in *wild type* (wt; *n* = 18) and *Emilin-2*^*−/−*^ (E2^−/−;^ *n* = 21) animals upon AOM/DSS induced CRC; each dot represents a single animal. **G** Graph showing the diameter of the colon lesions developed in *wild type* (wt) and *Emilin-2*^*−/−*^ (E2^−/−^) mice; each dot represents a single tumor. **H** Evaluation of the tumor diameter of the CRC lesions developed in *wild type* (wt) and *Emilin-2*^*−/−*^ (E2^−/−^) mice. Graphs in C, F and g represent the mean ± SD; *P* values were obtained using the paired Student’s t-test; **P < 0.01, ***P < 0.001, n.s.: *P* > 0.05

To investigate the effects of EMILIN-2 loss in CRC development, we took advantage of the *Emilin-2*^*−/−*^ mouse model [35]. First, we assessed the expression of EMILIN-2 in the colonic mucosa and, in *wt* animals, we found a strong EMILIN-2 signal within the lamina propria surrounding the crypts (Fig. 2A). As expected, no signal was found in *Emilin-2*^*−/−*^ mice.

To verify if the lack of EMILIN-2 could affect CRC development, we chemically-induced CRC in *wt* and *Emilin-2*^*−/−*^ mice by means of the AOM/DSS protocol. The AOM/DSS treatment dramatically impacted on the survival of *Emilin-2*^*−/−*^ mice due to excessive intestinal dysfunction resulting in chachexia (Fig. 2B). Mice were monitored weekly and the disease activity index (DAI) was calculated based on weight loss, intestinal bleeding and stool consistency. The DAI indicated that *Emilin-2*^*−/−*^ mice displayed more severe signs of suffering compared to *wt* animals (Fig. 2C). To assess tumor growth over time, mice were monitored by endoscopy; small polyps were detectable 10 weeks after the beginning of the treatment (Fig. 2D). At 25 weeks from the AOM injection, neoplastic lesions were macroscopically visible in the terminal part of the intestinal tract (Fig. 2E). In agreement with our hypothesis that the absence of EMILIN-2 may result in an increased activation of Wnt/β-catenin signaling pathway, one of the major drivers of CRC growth [29], *Emilin-2*^*−/−*^ mice developed a significant higher number of tumors compared with the *wt* littermates (Fig. 2D,F). Notably, only 65% of the *wt* mice developed CRC, while all the *Emilin-2*^*−/−*^ animals displayed one or more tumors (Fig. 2F). The tumor burden was comparable between the two mouse models (Fig. 2G), even if the *Emilin-2*^*−/−*^ mice were characterized by a slightly increased number of lesions with a diameter higher than 0.3 cm, despite the differences were not significant (Fig. 2H).

### The exacerbated CRC development in *Emilin-2*^*−/−*^ mice does not hinge on an altered Wnt/β-catenin signaling

Since we had previously shown that EMILIN-2 attenuates the Wnt/β-catenin signaling pathway in breast cancer cells [29], we next verified if tumors from *Emilin-2*^*−/−*^ mice displayed an increased activation of this pathway. Unexpectedly and in contrast to our hypothesis, in tumors from *wt* and *Emilin-2*^*−/−*^ mice the levels of active β-catenin, as well as the expression of the target genes c-Myc and cyclin D1, were comparable (Fig. S3A, B). Consistently, cell proliferation was similar in tumors developed in the two mouse models (Fig. S3C).

We next hypothesized that EMILIN-2 could influence colorectal cancer cell behavior impinging on other molecular mechanisms. To verify this hypothesis, we used human HT-29 and HCT-116 CRC cells, which do not express EMILIN-2 (Fig. S4A, B). Contrary to our hypothesis, the treatment with soluble recombinant EMILIN-2 did not affect their viability or apoptotic rate (Fig. S4C, D), nor their capability to form colonies in 3D settings (Fig. S4E, F). These results excluded the possibility of a direct effect of EMILIN-2 on colon cancer cell growth. However, EMILIN-2 inhibited the migration of HCT-116 cells, despite the migration rate of these cells was modest (Fig. S4G), whereas HT-29 cells failed to migrate with or without the treatment (data not shown). These results suggested that EMILIN-2 could directly affect the migration of colon cancer cells rather than the proliferation, and may impinge on metastatic dissemination.

### EMILIN-2 impacts on CRC formation affecting the inflammatory response

**Fig. 3:**
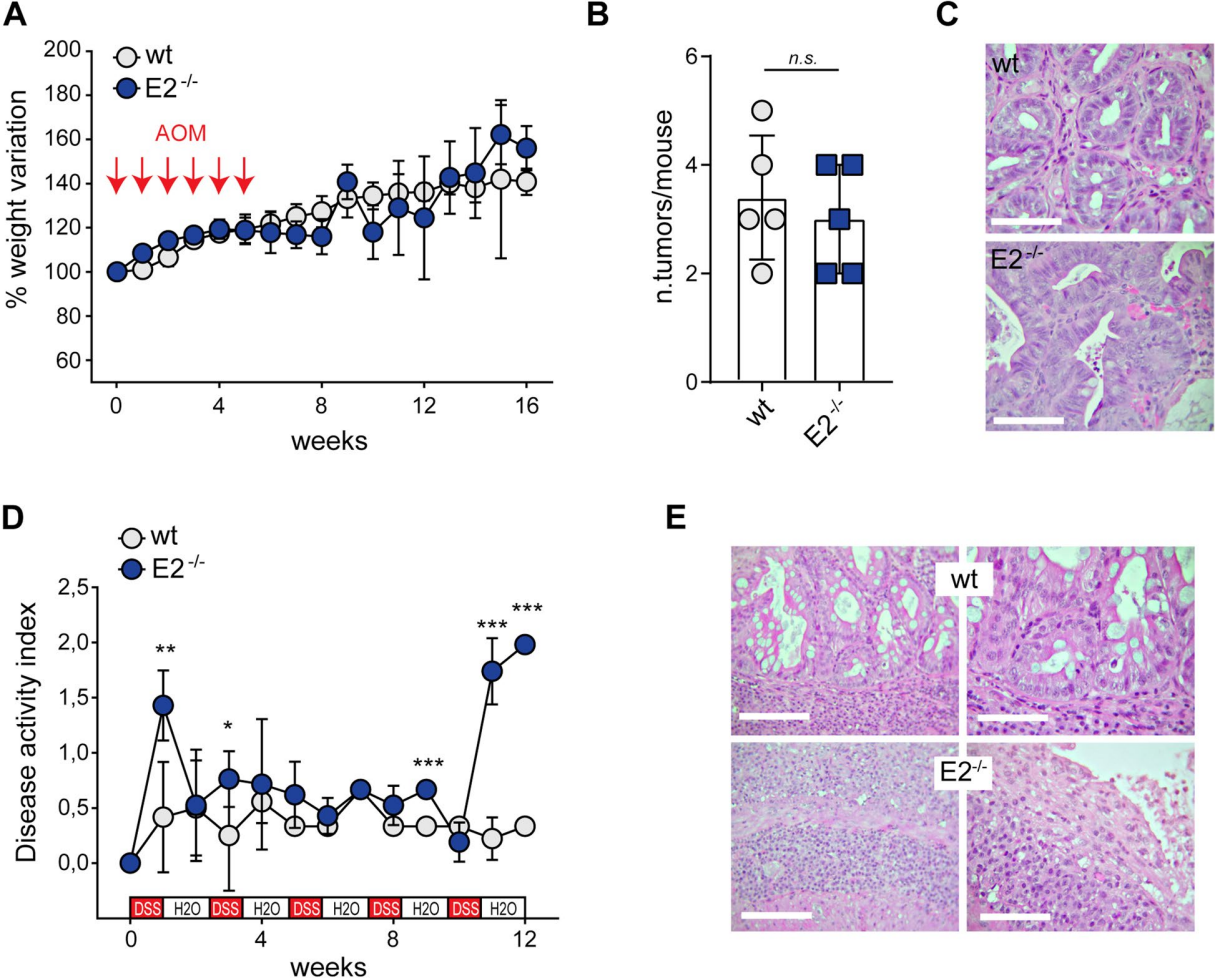
Ablation of EMILIN-2 associates with a harsh inflammatory response. **A** Graph showing the weight of *wild type* (wt) and *Emilin-2*^*−/−*^ (E2^−/−^) mice during the establishment of a sporadic model of CRC following 6 injections of AOM (red arrows), *n* = 5. **B** Graph indicating the number of colonic lesions present in *wild type* (wt) and *Emilin-2*^*−/−*^ (E2^−/−^) mice after 16 weeks from the first AOM injection. **C** Representative H&E images of the tumors developed in *wild type* (wt) and *Emilin-2*^*−/−*^ (E2^−/−^) mice as in B; magnification 400x; scale bar = 50 μm. **D** Graph representing the Disease Activity Index (DAI) of *wild type* (wt) and *Emilin-2*^*−/−*^ (E2^−/−^) mice during repeated cycles of DSS administration (red boxes), *n* = 5. **E** Representative H&E images of severe colitis in *wild type* (wt) and *Emilin-2*^*−/−*^ (E2^−/−^) mice after 12 weeks from the first DSS treatment. Magnification 400x; scale bar = 50 μm. Graphs in A, B and D represent the mean ± SD; *P* values were obtained using the paired Student’s t-test; * *P* < 0.05, **P < 0.01, ***P < 0.001, n.s.: *P* > 0.05

Ruled out the possibility of a direct action of EMILIN-2 in CRC cell growth, based on the fact that the protocol to induce CRC carcinogenesis included the pro-inflammatory agent DSS, we next hypothesized that the increased tumor growth in *Emilin-2*^*−/−*^ mice upon AOM/DSS treatment could depend on an altered inflammatory response. We thus dissected the contribution of the single components administrating either AOM alone or, alternatively, DSS alone to establish a model of sporadic or chronic DSS-induced inflammatory CRC, respectively (Fig. 3).

During AOM treatment, the animals did not show signs of systemic disease, in fact loose stools or bleeding were not detected and their weight increased physiologically (Fig. 3A). At 16 weeks from the first AOM injection, colorectal samples from *wt* and *Emilin-2*^*−/−*^ mice displayed no significant difference in the number of tumor lesions (Fig. 3B). Both mouse models developed tubular adenomas, tubulo-villous adenomas and adenocarcinomas. Nonetheless, the presence of adenomas displaying villous histological features or severe epithelial dysplasia, both associated to a greater propensity to develop carcinomas, were more frequent in *Emilin-2*^*−/−*^ mice (Fig. 3C).

On the contrary, DSS administration alone failed to induce the formation of CRC lesions in both *wt* and *Emilin-2*^*−/−*^ mice; however, the treatment was stopped at 12 weeks following the first DSS treatment, instead of the scheduled 16 weeks, due to severe suffering of the *Emilin-2*^*−/−*^ mice. As observed upon AOM/DSS administration, following prolonged DSS treatment *Emilin-2*^*−/−*^ animals were characterized by a much higher DAI compared to *wt* littermates (Fig. 3D). After 12 weeks, the colonic mucosa of both *wt* and *Emilin-2*^*−/−*^ mice showed massive and diffuse inflammation with ulcerative lesions, crypt abscesses and glandular architectural distortion (Fig. 3E). In addition, the overall condition of the colonic mucosa was poorer in *Emilin-2*^*−/−*^ mice; the ulcerative lesions were wider, occasionally resulting in necrotic-hemorrhagic foci of the lamina propria, and in large tracts of the mucosa, the crypt epithelium was replaced by granulation tissue (Fig. 3E). In few *Emilin-2*^*−/−*^ mice, the colon progressively swelled and became gangrenous developing toxic megacolon.

In order to determine if EMILIN-2 expression could be impaired not only during CRC growth, but also in the context of chronic inflammation, we interrogated a cohort of 363 patients affected by chronic intestinal inflammation (GEO ID: GSE83687) [52]. These analyses indicated that *EMILIN-2* gene is down-modulated also in the context of inflammatory bowel diseases (IBD) (Fig. S5). This result suggests that loss of EMILIN-2 may also occur before the IBD-associated CRC onset, thus creating a favorable soil for CRC development.

### The colonic mucosa from *Emilin-2*^*−/−*^ mice treated with DSS displays an altered myeloid cell infiltration

**Fig. 4:**
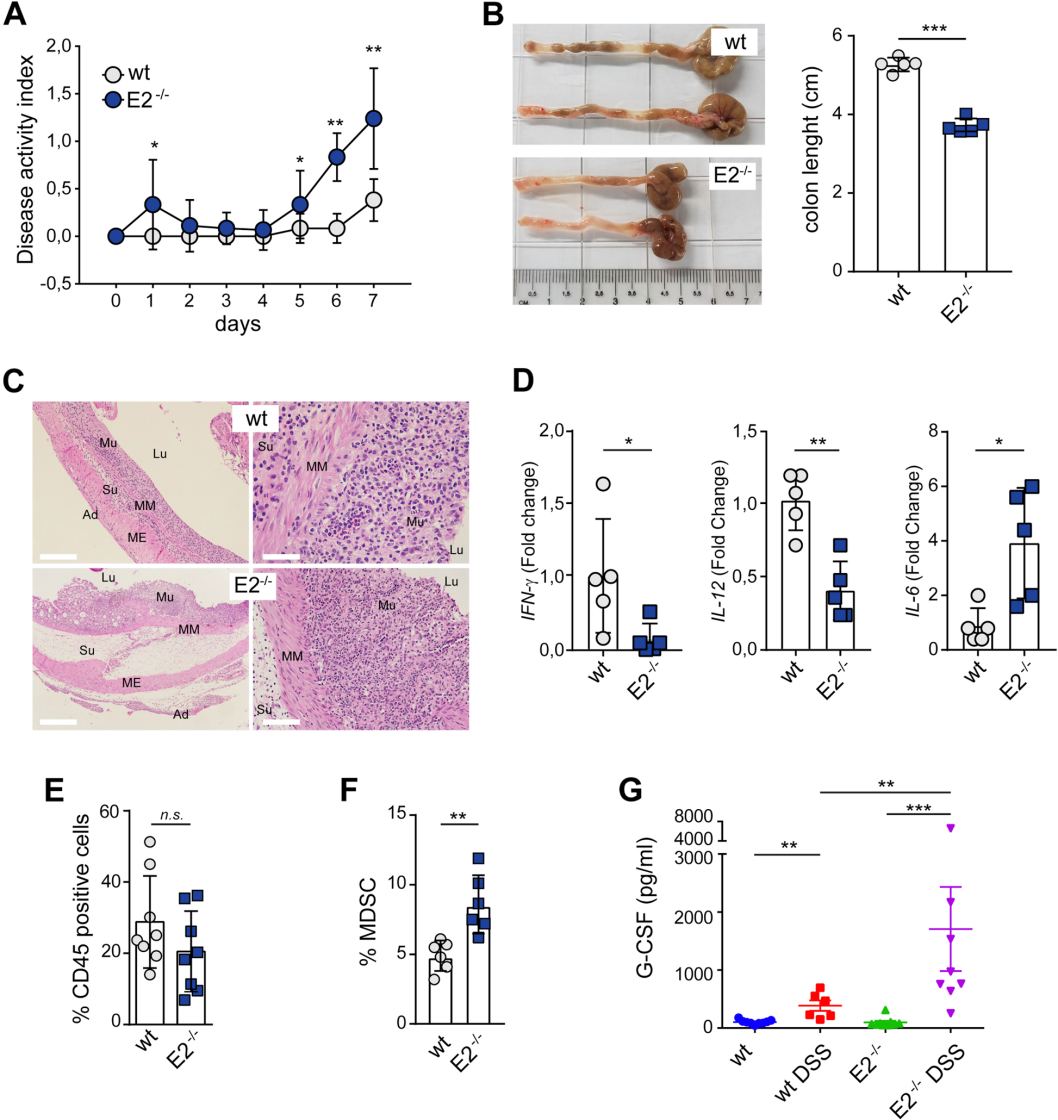
EMILIN-2 loss associates with an immune supressive microenvironment. **A** Graph showing the Disease Activity Index (DAI) of *wild type* (wt) and *Emilin-2*^*−/−*^ (E2^−/−^) mice during DSS administration, *n* = 5. **B** Representative images and graph reporting the length of *wild type* (wt) and *Emilin-2*^*−/−*^ (E2^−/−^) colons at day 7 of the experiment reported in A. **C** Representative H&E images of the colonic mucosa of *wild type* (wt) and *Emilin-2*^*−/−*^ (E2^−/−^) mice after 7 days of DSS administration; magnification: left images 50x (scale bar = 500 μm); right images: 200x (scale bar = 125 μm). Lu: Lumen; Mu: Mucosa; MM: Muscularis mucosa; Su: Submucosa; ME: Muscularis externa; Ad: Adventitia. **D** qPCR evaluation of the expression of *Interferon-γ* (*IFN-γ*), *Interleukin-12* (*IL-12*) and *Interleukin-6* (*IL-6*) relative to that of *GAPDH*, in colonic samples from *wild type* (wt) and *Emilin-2*^*−/−*^ (E2^−/−^) mice after 7 days of DSS administration; *n* = 5. **E** Flow cytometry analyses of total leukocytes (% CD45^+^/total cells) infiltrating the *wild type* (wt) and *Emilin-2*^*−/−*^ (E2^−/−^) colonic mucosa after 7 days of DSS administration, assessed as percentage of CD45 positive cells; *n* = 8. **F** Evaluation of total Myeloyd Derived Suppressor Cells (MDSC) in the *wild type* (wt) and *Emilin-2*^*−/−*^ (E2^−/−^) colonic mucosa after 7 days of DSS administration, assessed by flow cytometry as percentage of CD11b^+^Gr1^+^ cells on CD45 positive cells; *n* = 6. **G** Graph indicating the concentration of G-CSF in sera from *wild type* (wt) and *Emilin-2*^*−/−*^ (E2^−/−^) mice treated or not with DSS for 7 days; *n* = 8. Graphs represent the mean ± SD; in A, B, D, E and F, *P* values were obtained using the paired Student’s t-test; in G, *P* values were obtained with the One Way Anova Test; * *P* < 0.05, **P < 0.01, ***P < 0.001, n.s.: *P* > 0.05

Since the AOM/DSS treatment associated with an increased number of neoplastic lesions in *Emilin-2*^*−/−*^ mice, we hypothesized that the lack of EMILIN-2 could lead to poor tumor immunosurveillance, leading to the escape of a higher number of transformed cells. To verify this hypothesis, we analyzed the acute phase of the colonic inflammatory response. Similarly to what observed under chronic DSS treatment (Fig. 3D), during the acute phase of the DSS-induced inflammation, the *Emilin-2*^*−/−*^ mice were characterized by higher weight loss, increased presence of loose stools and bleeding, compared to *wt* animals (Fig. 4A). Additionally, the *Emilin-2*^*−/−*^ mice displayed reduced colon length, a feature associated to extensive phlogosis (Fig. 4B). Accordingly, inflammation and edema were more pronounced in *Emilin-2*^*−/−*^ mice and also infiltrated the submucosa and the adventitia (Fig. 4C).

Since IFN-γ, IL-12 and IL-6 are key regulators of the inflammatory response [53, 54], we verified the expression of these molecules in the colonic mucosa of the treated mice. Notably, the expression of IFN-γ and IL-12 was lower in *Emilin-2*^*−/−*^ mice compared to *wt* littermates, whereas that of IL-6, which associates with poor CRC prognosis [55], was higher (Fig. 4D).

Having determined an alteration of the inflammatory cytokines in the two mouse models, we next verified if this associated with an altered recruitment of inflammatory cells. First, we examined the leukocytes content and found that the total number of leukocytes infiltrating the intestinal tract was slightly, but not significantly lower in *Emilin-2*^*−/−*^ mice (Fig. 4E). Next, we hypothesized that the increased number of tumor lesions observed in *Emilin-2*^*−/−*^ mice could be accounted for the escape of tumor cells from immunosurveillance. Indeed, the number of myeloid derived suppressors cells (MDSC), whose presence associates with poor patient prognosis [56, 57], was significantly higher in *Emilin-2*^*−/−*^ mice (Fig. 4F). Taken together, these results indicate that the absence of EMILIN-2 associates with the onset of an immunosuppressive microenvironment.

To verify if the altered recruitment of inflammatory cells within the tumors was due to unbalanced levels of circulating immune cells, the number of circulating monocytes, lymphocytes and neutrophils was analyzed. Remarkably, as opposed to *wt* animals, the AOM/DSS-treatment did not induce any increase of monocytes, lymphocytes and neutrophils in the peripheral blood of *Emilin-2*^*−/−*^ mice (Fig. S6A). To assess if this was due to an altered maturation of the hematopoietic subpopulations, the cells were isolated from the bone marrow of wt and *Emilin-2*^*−/−*^ mice. These analyses excluded this possibility, since the bone marrow subpopulations were comparable (Fig. S6B).

Hence, we next determined if the abnormal immune cell recruitment could depend on altered cytokines/chemokines serum levels. In fact, the induction of acute inflammation in *wt* animals resulted in the increase of key inflammatory cytokines such as IFN-γ, GM-CSF, IL-4, IL-1β, and TNF-α (Fig. S7A). On the contrary, in *Emilin-2*^*−/−*^ mice the treatment did not alter the levels of IL-4, GM-CSF and TNF-α, instead it associated with lower levels of IFN-γ and IL-1β (Fig. S7A). Notably, the expression of the Granulocyte Colony Stimulating Factor (G-CSF), known to be associated with myeloid cell infiltration and linked to a worse outcome in AOM/DSS-treated mice [58], was strikingly higher with a 6-fold increase in *Emilin-2*^*−/−*^ mice (Fig. 4G). These alterations were also associated with a different pattern of CCL2 and CCL3 (Fig. S7B).

### EMILIN-2 loss associates with an altered inflammatory tumor microenvironment

**Fig. 5:**
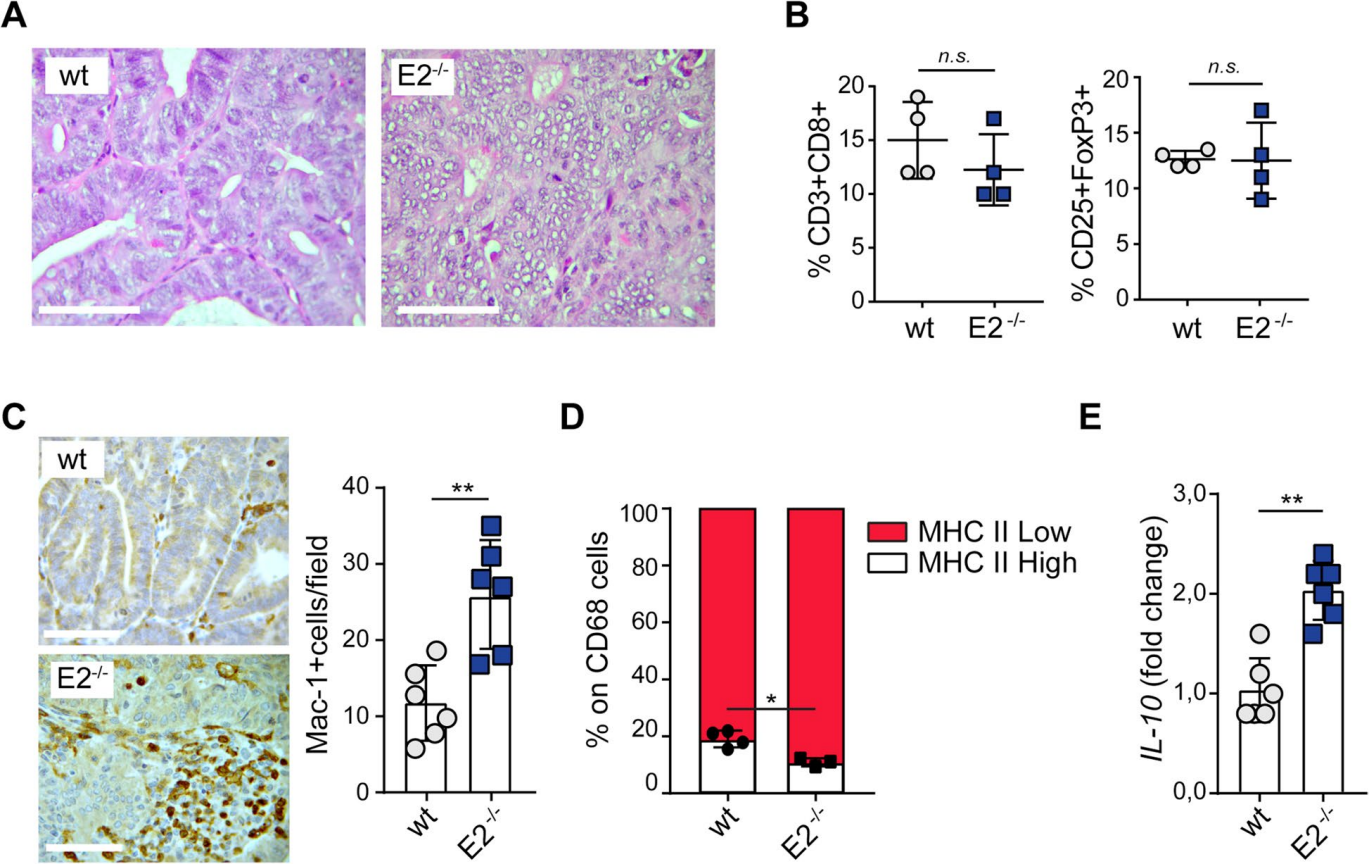
*Emilin-2*^*−/−*^ mice display an altered tumor associated inflammatory response. **A** Representative H&E images of tumors developed in *wild type* (wt) and *Emilin-2*^*−/−*^ (E2^−/−^) mice treated with AOM and DSS; magnification 400x; scale bar = 50 μm. **B** Analyses of cytotoxic T cells (left, CD3 + CD8+) and Treg cells (right, CD4^+^CD25^+^Foxp3^+^) infiltrating the tumors from *wild type* (wt) and *Emilin-2*^*−/−*^ (E2^−/−^) mice, assessed as percentage of CD3^+^ cells by flow cytometry; *n* = 4. **C** Representative images and quantification of macrophages (Mac-1 positive cells) infiltrating the tumors developed in *wild type* (wt) and *Emilin-2*^*−/−*^ (E2^−/−^) mice; *n* = 6; magnification 400x; scale bar = 50 μm. **D** Evaluation of the percentage of CD68^+^ cells expressing high or low levels of MHC II in tumors from *wild type* (wt; *n* = 4) and *Emilin-2*^*−/−*^ (E2^−/−^; *n* = 3) mice, as assessed by flow cytometry. **E** qPCR evaluation of the expression of *Interleukin-10* (*IL-10*) relative to that of *GAPDH*, in tumors from *wild type* (wt) and *Emilin-2*^*−/−*^ (E2^−/−^) mice; *n* = 6. Graphs represent the mean ± SD; *P* values were obtained using the paired Student’s t-test; * *P* < 0.05, **P < 0.01, n.s.: *P* > 0.05

Overall, our observations indicated that tumors developed in *Emilin-2*^*−/−*^ mice were not only more frequent but were also characterized by histological features indicating a higher propensity to progress into adenocarcinomas. *Emilin-2*^*−/−*^ tumors displayed cellular pleomorphism and a poorly differentiated phenotype (Fig. 5A).

To examine if this pattern could be linked to an altered tumor-associated inflammation, we analyzed the inflammatory component associated with established tumors. These analyses indicated that the absence of EMILIN-2 did not affect the recruitment of cytotoxic T cell and Treg cells (Fig. 5B); however, tumors developed in *Emilin-2*^*−/−*^ mice displayed a significant higher number of mononuclear cells mainly consisting of macrophages (Fig. 5C and Fig. S8A). The massive presence of macrophages was observed in the AOM/DSS (colitis-associated CRC) model, as well as in the AOM-induced (sporadic) model (Fig. S8B), suggesting that EMILIN-2 affects not only the immunosurveillance in the early phases of CRC development, but also the immune environment in established tumors (Fig. 5C and Fig. S8B).

A rich macrophage infiltrate normally associates with a more favorable outcome [59], in contrast tumors grew more efficiently in *Emilin-2*^*−/−*^ mice, despite they displayed an extensive macrophage infiltrate. We thus and this was in contrast with our preclinical observations, we examined the quality of the macrophage infiltrate and indeed we verified that the balance tilted towards the tumor-promoting M2 phenotype. In fact, the macrophages infiltrating the tumors of *Emilin-2*^*−/−*^ mice were characterized by an altered M1/M2 ratio and displayed a lower number of cells expressing high levels of the M1 marker MHC II (Fig. 5D). The prevalence of M2 macrophages in the tumor tissues from *Emilin-2*^*−/−*^ mice was consistent with a 100% increase of IL-10 (Fig. 5E), one of the main M2-associated cytokines [60].

### EMILIN-2 affects the M1/M2 macrophage polarization by triggering TLR-4 activation

To further explore the effects of EMILIN-2 on macrophages’ behavior we employed the human monocytic cell line U937. Cells were used under basal conditions or upon TPA-induced differentiation into a macrophage-like phenotype. EMILIN-2 did not affect the TPA-induced activation of U937 cells, their apoptotic rate, nor proliferation (Fig. S9A-D) and did not represent a substrate for U937 cell adhesion or migration (Fig. S9E, F).

**Fig. 6:**
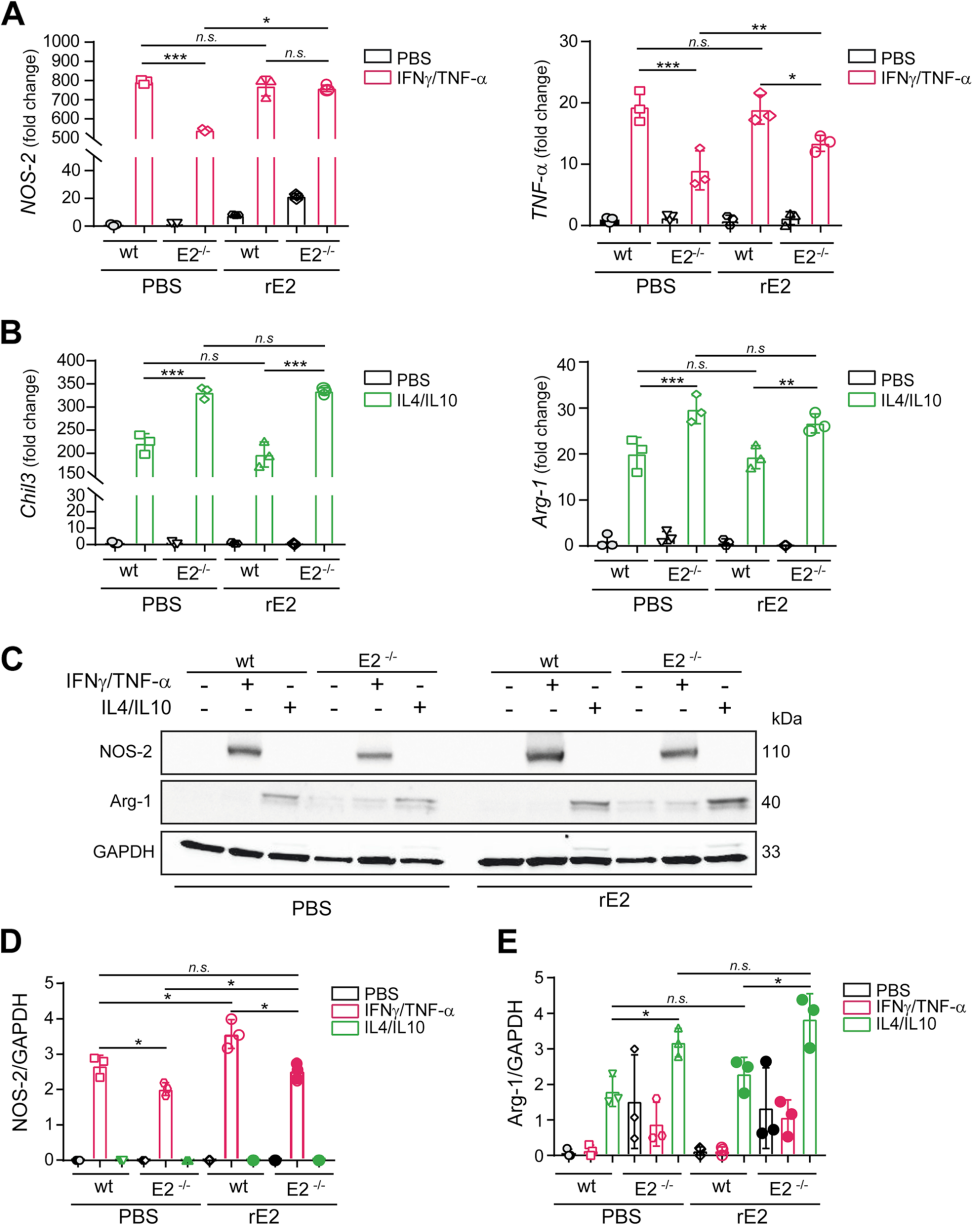
EMILIN-2 loss tilts the macrophage polarization towards the M2 phenotype. **A** qPCR analysis of the expression of the M1 markers *NOS-2* and *IL-1β*, relative to that of *GAPDH*, in bone marrow derived macrophages from *wild type* (wt) and *Emilin-2*^*−/−*^ (E2^−/−^) mice upon IFNγ/TNF-α treatment in the presence of recombinant EMILIN-2 (rE2) or vehicle (PBS); *n* = 4. **B** qPCR analysis of the expression M2 markers *Chil3* and *Arg-1*, relative to that of *GAPDH*, in bone marrow derived macrophages from *wild type* (wt) and *Emilin-2*^*−/−*^ (E2^−/−^) mice upon IL-4/IL-10 treatment in the presence of recombinant EMILIN-2 (rE2) or vehicle (PBS); *n* = 4. **C** Western blot analyses of the expression of NOS-2 and Arg-1 in bone marrow-derived macrophages from *wild type* (wt) and *Emilin-2*^*−/−*^ (E2^−/−^) mice upon treatments with IFNγ/TNF-α or IL-4/IL-10 in the presence of recombinant EMILIN-2 (rE2) or vehicle (PBS); *n* = 3. **D** and **E** Quantification of the results reported in C. Graphs represent the mean ± SD; *P* values were obtained using the One Way Anova Test; * *P* < 0.05, **P < 0.01, ***P < 0.001, n.s.: *P* > 0.05

To shed light on the role of EMILIN-2 in macrophage polarization, monocytes were isolated from *wt* and *Emilin-2*^*−/−*^ mice, differentiated into macrophages and stimulated towards the M1 or M2 phenotype (Fig. 6). Upon differentiation, the expression of the M1 markers Nos-2 and TNF-α was lower in macrophages derived from *Emilin-2*^*−/−*^ mice, suggesting that these cells were less prone to polarize towards the M1 phenotype (Fig. 6A). Importantly, treatment with recombinant EMILIN-2 partially improved the M1 polarization in macrophages derived from *Emilin-2*^*−/−*^ mice (Fig. 6A). On the contrary, upon treatment with IL-4 and IL-10, macrophages from *Emilin-2*^*−/−*^ mice expressed higher levels of the M2 makers Chil3 and Arg-1; however, treatment with recombinant EMILIN-2 did not further increase the levels of M2 markers in these cells (Fig. 6B). Similar results were obtained verifying the protein levels of NOS-2 and Arg-1 (Fig. 6 C-E).

**Fig. 7:**
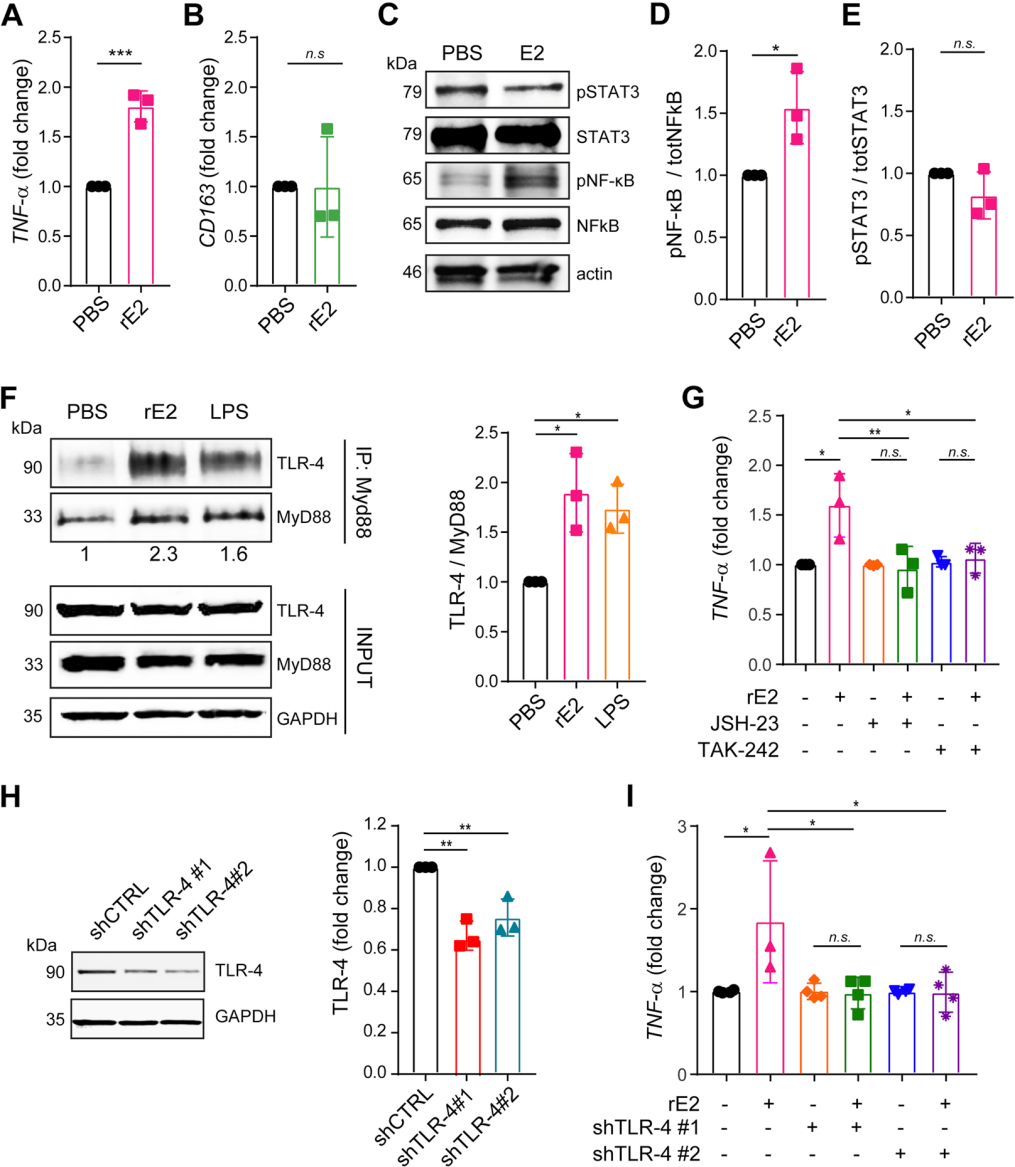
EMILIN-2 triggers TLR-4 activation promoting M1 polarization. **A** qPCR analyses of the expression of *TNF-α* (M1 marker) relative to *GAPDH* in TPA-activated THP-1 cells upon IFN-γ/LPS treatment in the presence of recombinant EMILIN-2 (rE2) or vehicle (PBS); *n* = 3. **B** qPCR analyses of the expression of *CD163* (M2 marker) relative to *GAPDH* in TPA-activated THP-1 cells upon IL-4 treatment in the presence of recombinant EMILIN-2 (rE2) or vehicle (PBS); *n* = 3. **C** Western blot analyses of pSTAT3 and pNF-κB in TPA-activated THP-1 cells challenged with recombinant EMILIN-2 (rE2) or vehicle (PBS); *n* = 3. **D** and **E** Quantification of the results reported in **C**. **F** Left: Western blot analyses of TLR-4 immunoprecipitation with the anti-MyD88 antibody using TPA-activated THP-1 cells challenged with recombinant EMILIN-2 (rE2), vehicle (PBS) or LPS as positive control. Whole lysates were loaded as input. Right: quantification of TLR-4 from three different experimental replicates. **G** qPCR analyses of the expression of *TNF-α* relative to *GAPDH* in TPA-activated THP-1 cells w/wo recombinant EMILIN-2 (rE2), and/or the TLR-4 (TAK-242) and NF-κB (JSH-23) inhibitors; *n* = 3. **H** Western blot (left) and quantification (right) of the down-regulation of TLR-4 upon lentiviral transduction with two specific shRNA constructs; pLKO served as a shRNA control (shCTRL). **I** qPCR analyses of *TNF-α* expression relative to that of *GAPDH* in TPA-activated THP-1 cells treated or not with recombinant EMILIN-2 (rE2) upon downregulation of TLR-4. Graphs represent the mean ± SD; *P* values were obtained using the paired Student’s t-test (A, B, D and E) or the One Way Anova Test (F, G, H and I); * *P* < 0.05, **P < 0.01, ***P < 0.001, n.s.: *P* > 0.05

To investigate the molecular mechanisms by which EMILIN-2 could affect macrophage polarization we employed the human THP-1 monocytic-like cell line. When challenged with EMILIN-2, TPA-activated THP-1 cells stimulated towards the M1 phenotype expressed higher levels of the M1 marker TNF-α (Fig. 7A), whereas, upon M2 stimulation, the expression levels of the M2 marker CD163 did not change (Fig. 7B), suggesting that also in this cell line EMILIN-2 tilts macrophages towards the M1 phenotype. We next analyzed the pathways involved in this response, assessing the activation of key downstream modulators of macrophage polarization, i.e. NF-κB and STAT1 for M1, and STAT3 for M2 [61]. EMILIN-2 stimulation resulted in increased pNF-κB levels (Fig. 7C, D), whereas the levels of pSTAT1 and pSTAT3 did not change (Fig. 7C, E and Fig. S10).

TLR-4 is a chief regulator of M1 polarization [61] and, upon engagement, it recruits MyD88 in the intracellular region, and the formation of this complex activates a signaling cascade leading to NF-κB phosphorylation. Thus, to verify if the increased EMILIN-2-dependent NF-κB phosphorylation hinged on TLR-4 activation, the TLR4/MyD88 complex was immunoprecipitated from TPA-activated THP-1 cells challenged with recombinant EMILIN-2 or the LPS positive control (Fig. 7F). In line with our hypothesis, EMILIN-2 significantly increased the levels of TLR-4 co-precipitating with MyD88, similarly to the effect of LPS, despite EMILIN-2 did not significantly alter TLR-4 expression (Fig. S11A, B). These results were corroborated by the use the TLR-4 and NF-κB inhibitors TAK-242 and JSH-23, respectively, which abolished the EMILIN-2-induced polarization towards the M1 phenotype, as assessed through the expression of the TNF-α marker (Fig. 7G). Overlapping results were obtained upon down-modulation of the TLR-4 expression by shRNA (Fig. 7H, I).

### EMILIN-2 affects the macrophages’ infiltrate and polarization in CRC patients

**Fig. 8:**
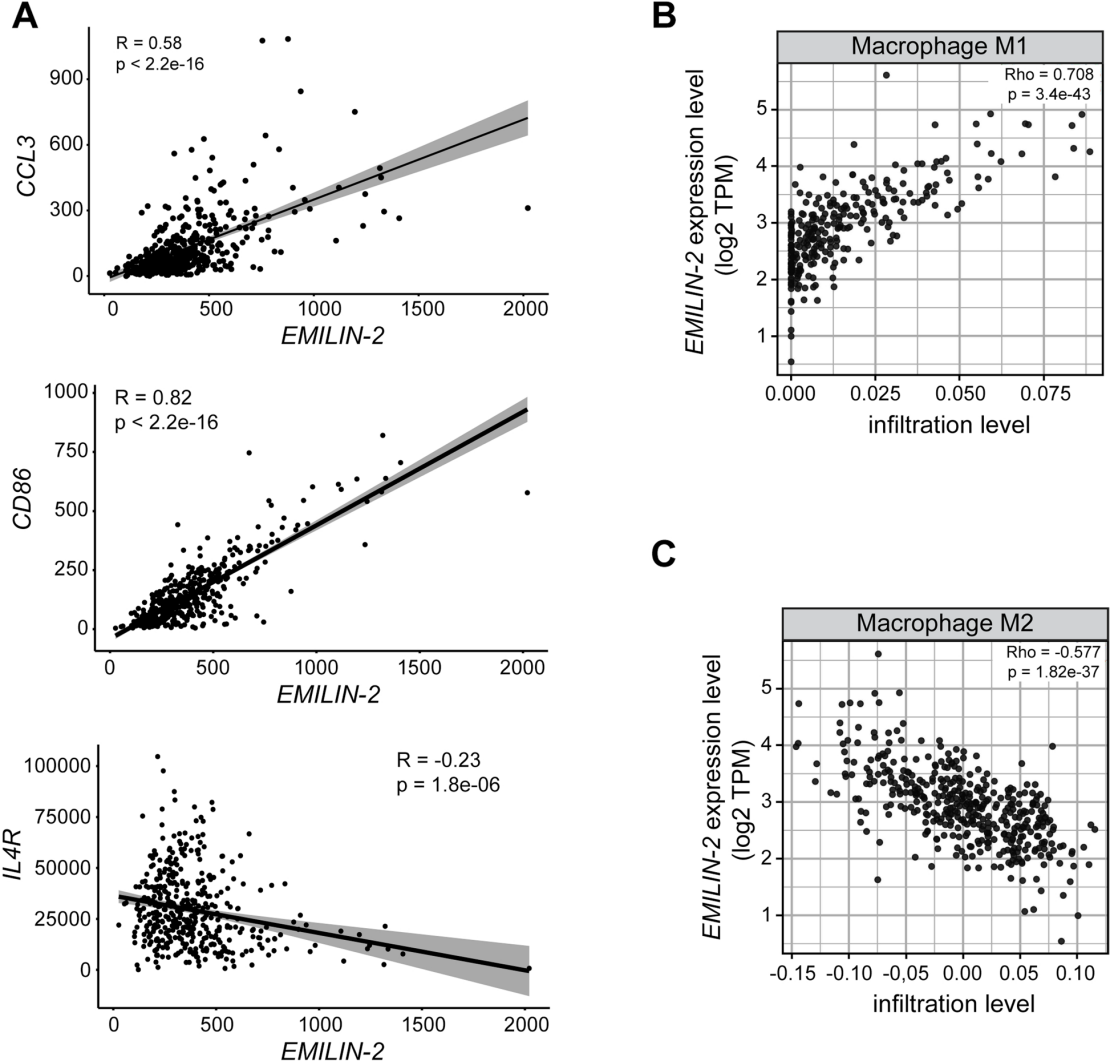
The levels of EMILIN-2 associate with macrophage polarization. **A** Spearman’s analysis correlating the expression levels of *EMILIN-2* with the macrophage markers (CCL3, CD86 and IL4R) in CRC patients from the TCGA COAD cohort (*n* = 477). **B** and **C** Spearman’s correlation analyses showing the association between *EMILIN-2* and the M1 or M2 macrophages in the same cohort reported in A, respectively. In B and C, the density of M1 or M2 cells was evaluated with the *timer2* tool. *P* < 0.05 values were considered statistically significant

Given the results obtained in vivo and in vitro, we next aimed at verifying if the levels of EMILIN-2 expression associated with the macrophages’ infiltrate and polarization also in CRC patients. To this end, we interrogated the TCGA COAD cohort and found that the levels of *EMILIN-2* positively correlated with the expression of the M1 markers CCL3 and CD86, and negatively correlated with the M2 marker IL4R (Fig. 8A). It must be pointed out that the tumor immune microenvironment is characterized by a remarkable complexity, thus a more comprehensive analysis was required to better define which parameters could better describe the relation between EMILIN-2 and macrophages. Among the numerous methods used to estimate immune cells from bulk RNAseq samples derived from the whole tumor [46], we took advantage of TIMER 2.0 resource, which allows a systematical analysis of the immune infiltrate using specific gene signatures. Importantly, in agreement with the data obtained in the in vivo mouse model, we identified a positive association between EMILIN-2 and the M1 macrophage infiltrate, whereas the correlation with the M2 macrophages was inverse (Fig. 8B, C). These results were further confirmed on the TGCA COAD cohort using the recently developed *immunodecov* tool (*P* < 2.2e-16 for both M1 and M2).

**Fig. 9:**
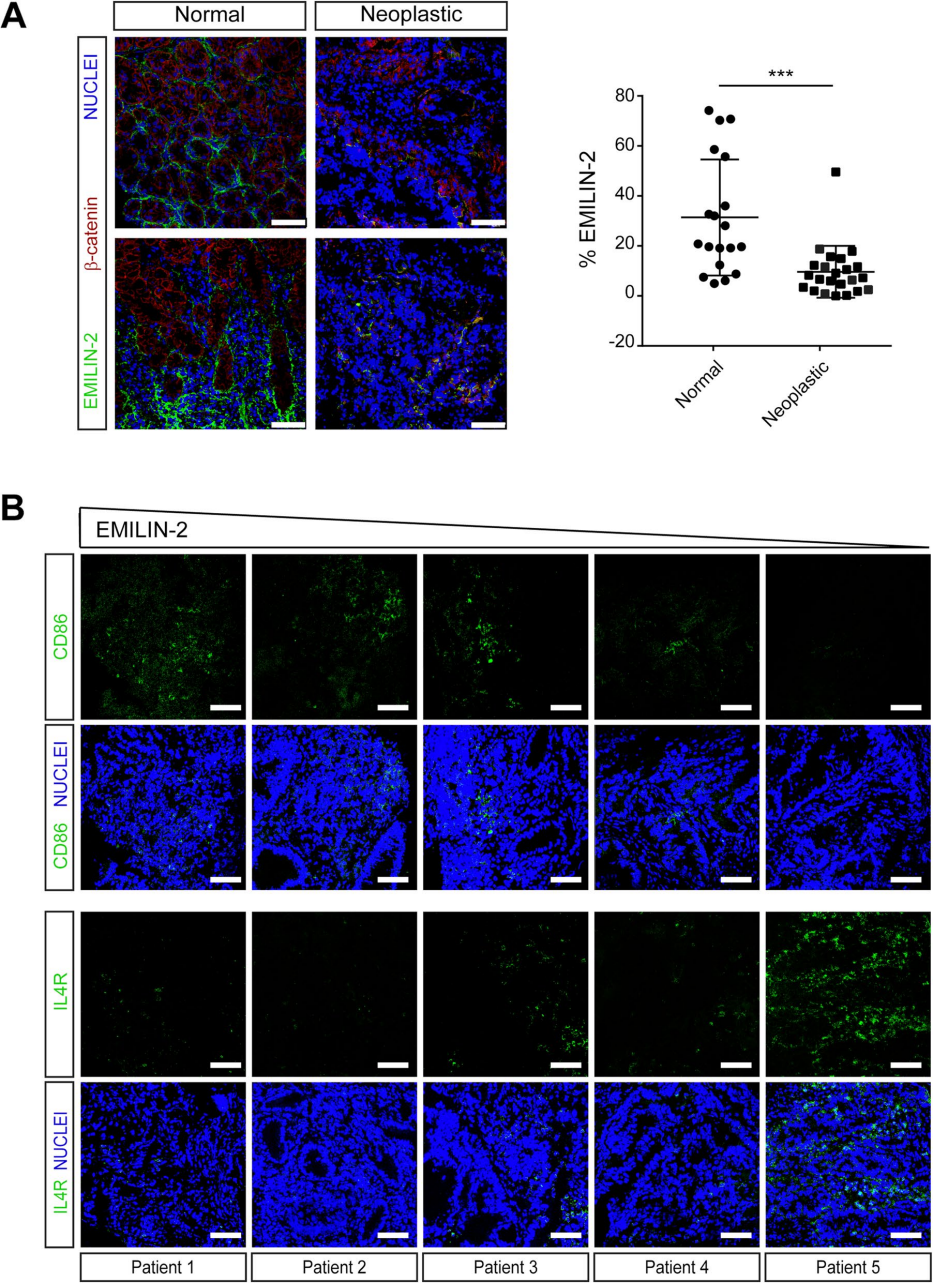
Loss of EMILIN-2 associates with low CD86 and high IL4R levels. **A** Representative images and quantification of the immunofluorescence analyses of EMILIN-2 in the bioptic samples of the normal colonic mucosa and the adjacent neoplastic tissue. Green: EMILIN-2, red: β-catenin, Blue: nuclei. Scale bar = 50 μm. **B** Representative images of the immunofluorescence analyses of CD86 (green, top panel) and IL4R (green, low panel) in five patients displaying different EMILIN-2 expression levels (from high to low, as indicated). Blue: nuclei. Scale bar = 50 μm. Graph represents the mean ± SD; *P* values were obtained using the paired Student’s t-test; ***P < 0.001

**Fig. 10:**
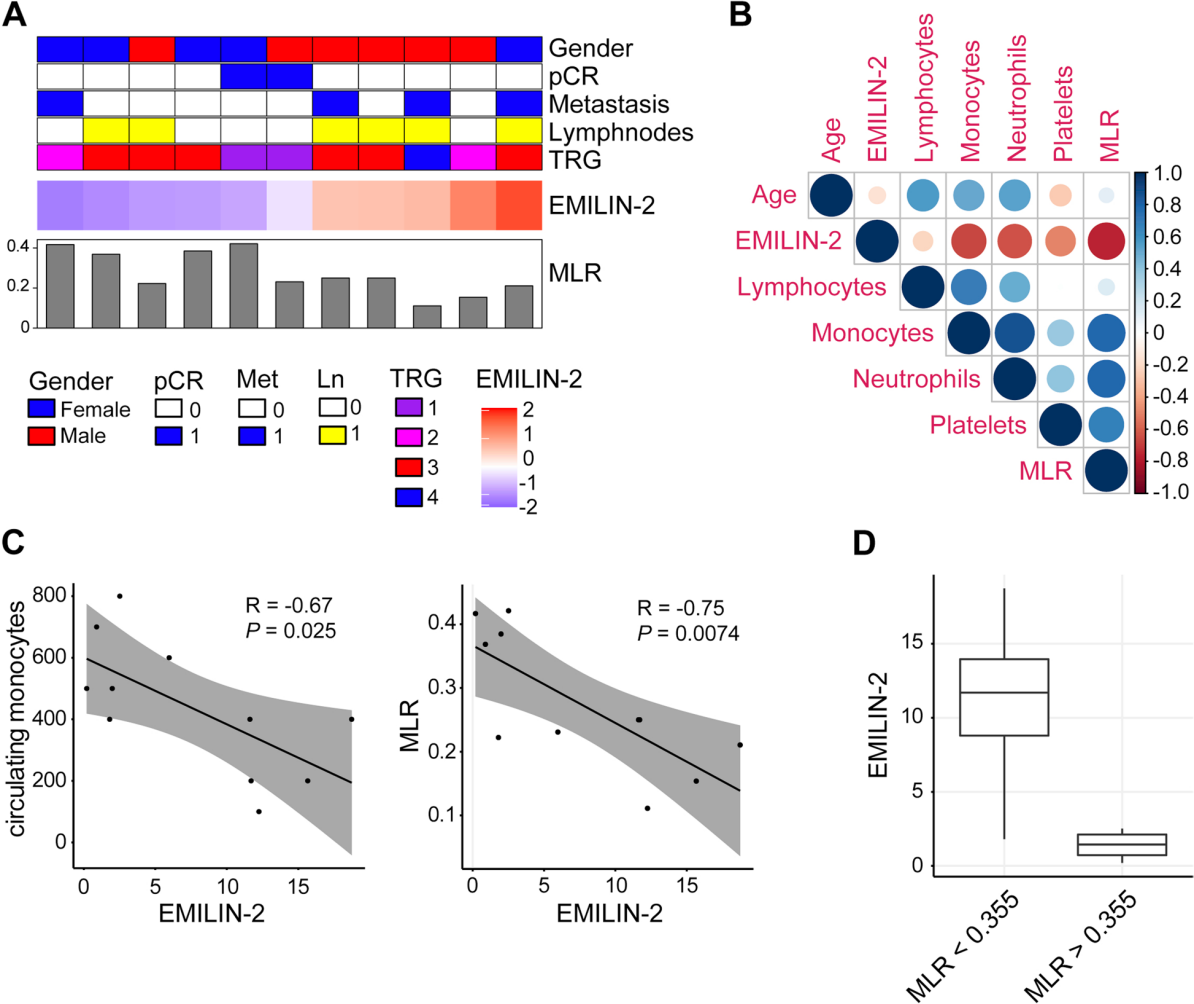
The EMILIN-2 levels correlate with the monocyte/lymphocyte ratio. **A** Stratification of the CRC patients cohort examined in our Institute based on the EMILIN-2 levels; the clinical parameters available for 11 patients are indicated. pCR: pathological complete response; TRG: tumor regression grade; MLR: monocyte/lymphocyte ratio. **B** Correlation matrix showing the association between the immune circulating cells and the EMILIN-2 levels in the patient cohort reported in A. Positive correlations are highlighted in blue and negative in red, color intensity and size of the circles being proportional to the correlation coefficients; MLR: monocyte/lymphocyte ratio. **C** Pearson correlation analyses of the levels of EMILIN-2 and the number of circulating monocytes (left) and the monocyte/lymphocyte ratio (MLR, right) in the patient cohort reported in A. **D** Levels of EMILIN-2 in CRC patients following stratification based on the monocyte/lymphocyte ratio (MLR) value (threshold = 0.355)

To verify the results obtained with the in-silico analyses, we exploited 23 biopsies from CRC patients diagnosed and treated in our Institute (Table S1). Bioptic samples were collected from normal and neoplastic sites during diagnostic endoscopy and the EMILIN-2 expression levels evaluated by immunofluorescence. Nineteen normal colonic samples were available for the analyses and overall EMILIN-2 was highly expressed and decorated the normal stroma surrounding the crypts (Fig. 9A). On the contrary, the tumor tissues were characterized by a strong reduction of the EMILIN-2 levels (Fig. 9A). Importantly, tumors displaying high EMILIN-2 levels were characterized by a high number of cells expressing the M1 markers CD86 and HLA-DR, as opposed to those displaying low levels of EMILIN-2, where the presence of IL-4R- and CD163-positive M2 cells was more pronounced (Fig. 9B and Fig. S12). Of these 23 CRC patients, only for 11 the clinical parameters and the complete blood count were available (Fig. 10A). Thus, for these patients we compared the tumor-associated EMILIN-2 expression levels with the blood count. Despite the low number of patients, EMILIN-2 strongly associated with the number of circulating inflammatory cells also in this small cohort (Fig. 10B). Interestingly, the number of circulating monocytes and, notably, the monocyte/lymphocyte ratio (MLR), which is a predictor of the patients’ outcome [62], negatively correlated with EMILIN-2 (Fig. 10C). It is known that patients characterized by a MLR < 0.355 have a better prognosis [62]. Consistently, in the 11 patients cohort from our Institute patients displaying a MLR value lower than 0.355 were characterized by higher EMILIN-2 levels, whereas patients with a MLR higher than 0.355 showed a 6-fold reduction of EMILIN-2 (Fig. 10D).

### Loss of EMILIN-2 correlates with a poor outcome of CRC patients

**Fig. 11:**
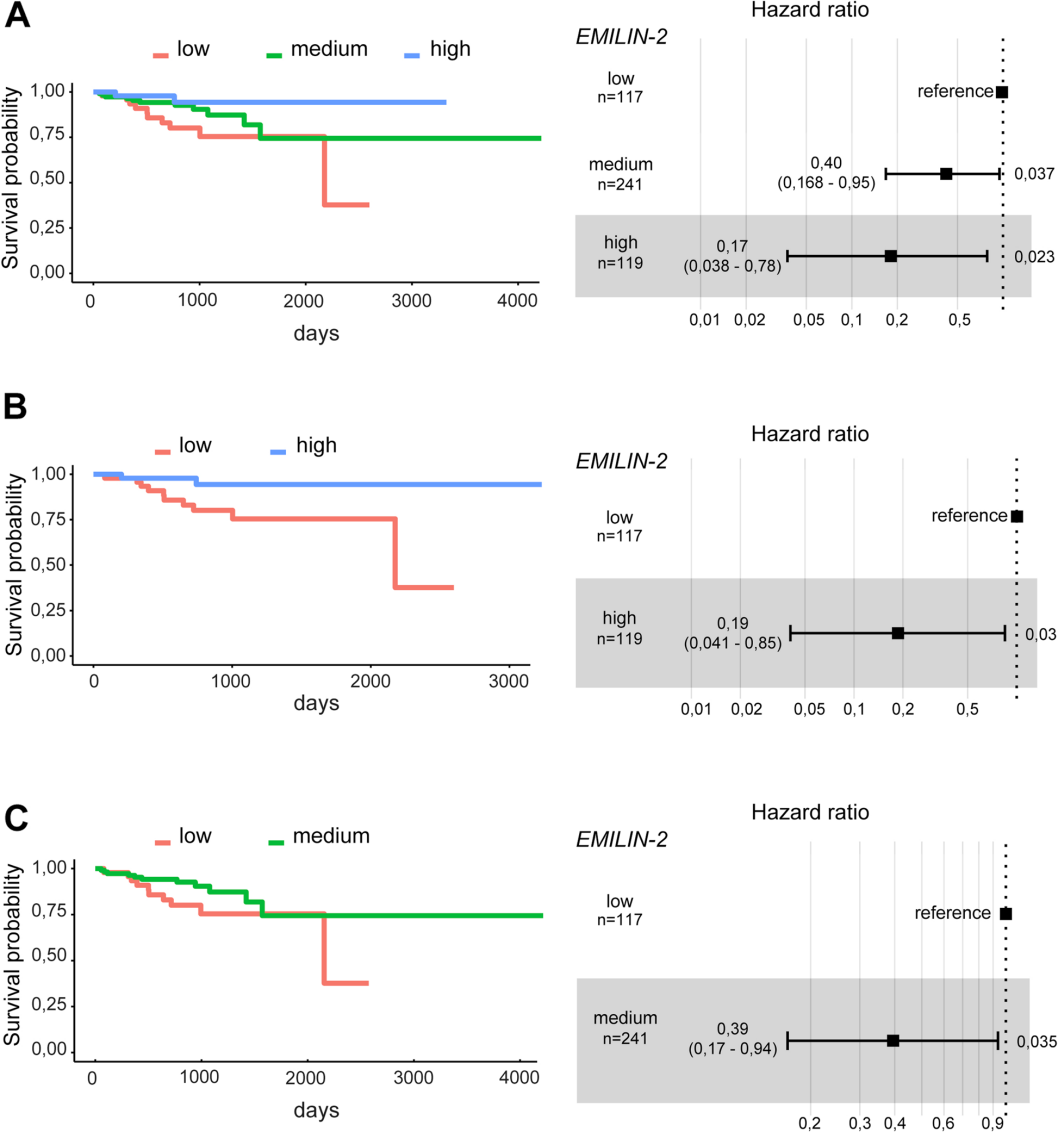
High EMILIN-2 expression levels associate with favorable CRC patient outcome. **A** Kaplan-Meier curves reporting the disease free survival of the COAD TCGA cohort including 477 CRC patients, stratified according to high, medium and low *EMILIN-2* expression levels. **B** and **C** Disease free survival of the same cohort stratified according to high and low or medium and low *EMILIN-2* expression levels, respectively. The Hazard ratios are reported on the right. Kaplan-Meier curves were computed using R (version 3.6.1) with the *survival* and *survminer* packages and compared with the log-rank test

Given these observations, we finally assessed if the *EMILIN-2* expression could associate with the outcome of CRC patients. To this end we evaluated the disease free survival (DFS) exploiting the TCGA COAD cohort [45]. Notably, as shown in Fig. 11A, loss of *EMILIN-2* associated with a lower DFS, and a moderate expression of *EMILIN-2* correlated with intermediate DFS values (Fig. 11A); more importantly, both high and moderate expression of *EMILIN-2* associated with a significantly better outcome of the CRC patients (Fig. 11B, C). Taken together these results pinpoint EMILIN-2 as an important regulator of the tumor inflammatory microenvironment and suggest that EMILIN-2 could represent a promising prognostic marker for CRC patients.

## Discussion

Patients affected by inflammatory bowel diseases are at higher risk of developing cancer and a vast amount of evidence indicate that spontaneous colon cancer is driven by inflammation, thus making this type of tumor a paradigm for the link between chronic inflammation and tumorigenesis [63–66]. CRC development is characterized by profound changes of the immune cell infiltrate and inflammatory cytokines expression, as well as alterations of the ECM [67]. However, researchers have only scratched the surface towards the understanding of the role of ECM in affecting the immune response. In this study, for the first time we highlight the prominent role of EMILIN-2 in regulating MDSC recruitment and macrophage polarization during CRC development.

EMILIN-2 acts as a tumor suppressor in different cancer types [27–29] and the finding that the molecule was down-regulated in CRC patients prompted us to hypothesize that it could play a crucial role in this context. Indeed, in a cohort of CRC patients, poor EMILIN-2 expression associated with increased levels of S100P [51], a marker of poor prognosis whose expression together with that of *APOBEC3G*, *CD133*, *LIPC* correlates with increased CRC hepatic metastasis [68].

Accordingly, *Emilin-2* null mice displayed increased susceptibility to colitis-associated tumorigenesis and worse outcome. However, here the effect on tumor growth was not regulated directly, as previously demonstrated in other tumor types, i.e. sarcomas or breast [27, 29]. In fact, the proliferation and apoptotic rate of tumor cells grown in *Emilin-2*^*−/−*^ mice was not altered, consistent with the results obtained using human colon cancer cells challenged with EMILIN-2. In accordance with these findings, the increased CRC growth in *Emilin-2*^*−/−*^ mice did not hinge on the Wnt/β-catenin signaling. It is conceivable to hypothesize that the regulation of the Wnt ligands’ availability exerted by EMILIN-2 [29] may be irrelevant when the *APC* and/or *CTNNB1* genes, the major targets of AOM [39], are constitutively hyperactive. This further highlights the complex regulations wielded by EMILIN-2, which may lead to different effects depending on the peculiar molecular characteristics of the tumor cells and/or the tumor microenvironment. An indication of the mechanism by which EMILIN-2 affected CRC development in mice was suggested by the treatment itself, which included a pro-inflammatory agent. A more detailed analysis of the immune traits suggested that EMILIN-2 shapes both the early and late phases of the immune response.

Although it was not possible to verify similar changes in CRC patients, our preclinical data indicated that in the early phases of CRC development EMILIN-2 up-regulates the expression of IL-12 and INF-γ, key cytokines supporting a tumor-prohibitive microenvironment [69]. In addition, during inflammation also the levels of G-CSF, a key regulator of MDSC cell recruitment [70], were dramatically increased in *Emilin-2*^*−/−*^ mice. Consistently, ablation of EMILIN-2 associated with a higher infiltration of MDSC cells, negative regulators of the tumor immunosurveillance [71]. This resulted in increased number of tumor lesions, suggesting that a higher expression of EMILIN-2 in the tumors may suppress the recruitment of MDSC cells restraining the escape of transformed tumor cells and the formation of CRC lesions. Escape of tumor cells from immunosurveillance not only allows the development and growth of primary tumors, but also the metastatic spread, which is the main cause of cancer-related death [72]. Despite the AOM/DSS-induced lesions hardly ever progresses to metastatic dissemination, in future studies it will be necessary to verify if EMILIN-2 also plays a role in this process.

Alteration of macrophages in *Emilin-2*^*−/−*^ mice were observed both in the sporadic and in colitis associated CRC models. The higher macrophage infiltration present in tumors developed in *Emilin-2*^*−/−*^ mice may be ascribed to low levels of IFN-γ. This cytokine is known to reduce macrophage motility but also to enhance the expression of pro-inflammatory genes [73]. Extensive macrophage infiltration associates with favorable patients’ outcome [74], however, an imbalance towards immune-suppressive and tumor-supporting macrophage phenotype can lead to poor outcome [75, 76]. This may explain why the *Emilin-2*^*−/−*^ mice displayed a higher number of tumor lesions despite the rich macrophage infiltrate. In agreement with an exacerbated CRC development, the tumor immune infiltrate in *Emilin-2*^*−/−*^ mice was characterized by an imbalance of the M1/M2 ratio, the polarization tilting towards the M1 phenotype. This finding was also confirmed using macrophages isolated from *wt* and *Emilin-2*^*−/−*^ mice. Mechanistically, we demonstrated that EMILIN-2 prompts M1 polarization by activating the TLR-4/MyD88/NF-κB signaling pathway. In CRC, the TLR-4 holds a prominent, yet controversial, role [61, 77, 78]. In fact, its expression in CRC cells associates with poor patients’ prognosis [78]. On the other hand, this study suggests that the activation of TLR-4 in tumor associated macrophages, which affects their polarization, is influenced by microenvironmental cues and may significantly impact on tumor growth and progression. Accordingly, database analyses of CRC patients demonstrated that loss of EMILIN-2 associated with an increased M2 macrophage infiltrate. *These results represent a novelty since to our knowledge no ECM molecule has been shown to regulate macrophage polarization prior to this study. N*onetheless, it is known that the specific composition of the ECM milieu from different tissues can influence the polarization towards an M1 or M2 phenotype [79]. In CRC, the switch of immune-stimulating M1 cells in favor of the immune-suppressive M2-like cells not only foster tumor growth and progression [80] but also promotes resistance to therapy [57, 81–84]; this possibility suggests that the expression levels of EMILIN-2 may also affect the efficacy of CRC treatment.

## Conclusions

Taken together, these results demonstrate that EMILIN-2 plays a dual indirect role in CRC development and growth by modulating the inflammatory microenvironment impacting on both the immunosurveillance and immunosubversion phases [14]. Differently from the cognate EMILIN1 molecule, which indirectly affects the immune microenvironment in CRC by acting on the lymphatic vasculature [85], EMILIN-2 influences immune cell activation via a direct mechanism involving the TLR-4 [86].

It is known that, under inflammatory conditions, the ECM landscape is altered by extensive remodeling/turnover, as well as dysregulated expression [87], processes that may also impact on the EMILIN-2 levels. Since the loss of EMILIN-2 is variable among the patients, the analysis of its expression may aid a patient-tailored prognosis. The potential of EMILIN-2 as a prognostic marker is supported by the finding that low levels of EMILIN-2 associate with poor prognosis, as assessed in a cohort of 477 CRC patients. However, these data are based on the *EMILIN-2* RNA levels. To verify the suitability of EMILIN-2 as a prognostic marker, it will be worth analyzing also the protein expression in a larger amount of samples. In addition, given the dual role of EMILIN-2 in angiogenesis and immune response, it would also be interesting to verify the levels of the protein in a cohort of patients undergoing anti-angiogenic therapy in combination with immunotherapy, and verify the correlation with the patients’ outcome. Taken together these observations further indicate that EMILIN-2 may represent a valuable prognostic biomarker for CRC patients.

## Supplementary Information

**Additional file 1: Table S1**.

**Additional file 2: Table S2**.

**Additional file 3: Figure S1**-**Figure S3**.

**Additional file 4: Figure S4**-**Figure S12**.

## Data Availability

Transcriptomics data from Cancer Genome Atlas of Colorectal Cancer type are available in the GDC Data portal (https://portal.gdc.cancer.gov/) and proteomics data are available from Vaaikar et al. using cBioportal (The cBio Cancer Genomics Portal: An Open Platform for Exploring Multidimensional Cancer Genomics Data). The expression profiles of genes in colon cancer patients are available at the GEO database (ID: GSE35834).

## Abbreviations

AOM: Azoxymethane
CCL2: C–C motif chemokine ligand 3
CCL3: C–C motif chemokine ligand 2
CRC: Colorectal cancer
DAI: Disease Activity Index
DFS: Disease free survival
DSS: Dextran Sodium Sulfate
ECM: Extracellular matrix
EGF: Epithelial growth factor
EGFR: Epithelial growth factor receptor
EMILIN-2: Elastin microfibril interface located protein 2 (protein)
*EMILIN-2*: Elastin microfibril interface located protein 2 (human gene name)
*Emilin-2*: Elastin microfibril interface located protein 2 (mouse gene name)
GM-CSF: Granulocyte-macrophage colony-stimulating factor
IFN-γ: Interferon-γ
IL-1β: Interleukine-1β
IL-6: Interleukine-6
IL-8: Interleukin-8
IL-10: Interleukin-10
IL-12: Interleukine-12
LPS: Lipopolysaccharides
MDSC: Myeloyd Derived Suppressor Cells
MLR: Monocyte/lymphocyte ratio
PD-1: Programmed cell death protein-1
PD-L1: Programmed death-ligand-1
TCGA: The Cancer Genome Atlas
TLR-4: Toll like receptor-4
TNF-α: Tumor Necrosis Factor-α
TPA: 12-O-Tetradecanoylphorbol-13-acetate
