# Supplementary material for: Colorectal cancer development is affected by the ECM molecule EMILIN-2 hinging on macrophage polarization via the TLR-4/MyD88 pathway
Source: J Exp Clin Cancer Res. 2022 Feb 11;41:60. doi: 10.1186/s13046-022-02271-y (PMC8840294; doi:10.1186/s13046-022-02271-y)
Supplement: Supplementary file 1 — Additional file 1: Table S1. [file 13046_2022_2271_MOESM1_ESM.pdf]

Table S1. Clinic-pathological characteristics of patients.

Variables	N. (23 tot)	%
**Sex**		
• M	12	52
• F	11	48
**Age**		
• <65	14	61
• >=65	9	39
**Stage pre-treatment**		
• II	6	26
• III	12	52
• IV	5	22
**Resection**	23	100
**Stage post-treatment**[1]		
• pCR	3	13
• II	4	17
• III	8	35
**Lymph involv. Post-treatment**[1]		
• No	7	30
• Yes	8	35
**TRG**[1]		
• 1	3	13
• 2	2	9
• 3	6	26
• 4	2	9
**pCR**[1]		
• No	10	43
• Yes	5	22
**Adj CT**[1]		
• No	6	40
• Yes	9	60
**Metastatic**[1]		
• No	10	43
• Yes	4	17
**Number of M. sites**[1]		
• 0	10	43
• 1	2	9
• 2	1	4
**Status**[1]		
• Alive	12	52
• Dead	2	9

[1]This information was not available for all the patients analyzed.
