# Supplementary material for: Colorectal cancer development is affected by the ECM molecule EMILIN-2 hinging on macrophage polarization via the TLR-4/MyD88 pathway
Source: J Exp Clin Cancer Res. 2022 Feb 11;41:60. doi: 10.1186/s13046-022-02271-y (PMC8840294; doi:10.1186/s13046-022-02271-y)
Supplement: Supplementary file 2 — Additional file 2: Table S2. [file 13046_2022_2271_MOESM2_ESM.pdf]

Table S2. Oligonucleotides used for quantitative PCR analyses.

Gene Name	Forward	Reverse
EMILIN-2	TTGAAGACATTTGCCTGCTG	AGTGAACCTACCCATGCCAG
hTLR-4	ACCTGTCCCTGAACCCTATGAA	CTTCTAAACCAGCCAGACCTTG
Ifngr1	TGCCTGTACCGACGAATGTT	TCCAGGAACCCGAATACACC
IL10ra	TGCATACGGGACAGAACTGC	CAGGACAATGCCTGAGCCTT
IL-12	GGAAGCACGGCAAGCAGAATA	AACTTGAGGGAGAGAAGTAGGAATGG
IL-1β	TGCCACCTTTTGACAGTGATG	TGATGTGCTGCTGCGAGATT
IL4ra	CCTCACACTCCACACCAATG	AGCCTGGGTTCCTTGTAGGT
mArg-1	GCTTGCTTCGGAACTCAACG	CTTGGGAGGAGAAGGCGTTT
mCCL2	AGCTGTAGTTTTTGTCACCAAGC	GTGCTGAAGACCTTAGGGCA
mCCL5	TGCTGCTTTGCCTACCTCTC	TCCTTCGAGTGACAAACACGA
mChil-3	ACTTTGATGGCCTCAACCTG	AATGATTCCTGCTCCTGTGG
mCMYC	CACCAGCAGCGACTCTGAA	CCCGACTCCGACCTCTTG
mCycD1	CATCAAGTGTGACCCGGACTG	CCTCCTCCTCAGTGGCCTTG
mGAPDH	AGGTCGGTGTGAACGGATTTG	TGTAGACCATGTAGTTGAGGTCA
mIFN-γ	CTTTGGACCCTCTGACTTGAG	TCAATGACTGTGCCGTGG
mIL-6	CAAAGCCAGAGTCCTTCAGAG	GTCCTTAGCCACTCCTTCTG
mNOS-2	CACCTTGGAGTTCACCCAGT	ACCACTCGTACTTGGGATGC
mTLR-4	AGATCTGAGCTTCAACCCCTTG	GGTGGTGTAAGCCATGCCA
mTNF- α	CTTCTGTCTACTGAACTTCGGG	CAGGCTTGTCACTCGAATTTTG
PD-1	TTCAGGTTTACCACAAGCTGG	TGACAATAGGAAACCGGGAA
PD-L1	GGAATTGTCTCAGAATGGTC	GTAGTTGCTTCTAGGAAGGAG
Tnfrsf1a	ACCGTGACAATCCCCTGTAA	GTCCTGGGGGTTTGTGACAT
