# Supplementary material for: Colorectal cancer development is affected by the ECM molecule EMILIN-2 hinging on macrophage polarization via the TLR-4/MyD88 pathway
Source: J Exp Clin Cancer Res. 2022 Feb 11;41:60. doi: 10.1186/s13046-022-02271-y (PMC8840294; doi:10.1186/s13046-022-02271-y)
Supplement: Supplementary file 3 — Additional file 3: Figure S1-Figure S3. [file 13046_2022_2271_MOESM3_ESM.pdf]

Figure S1

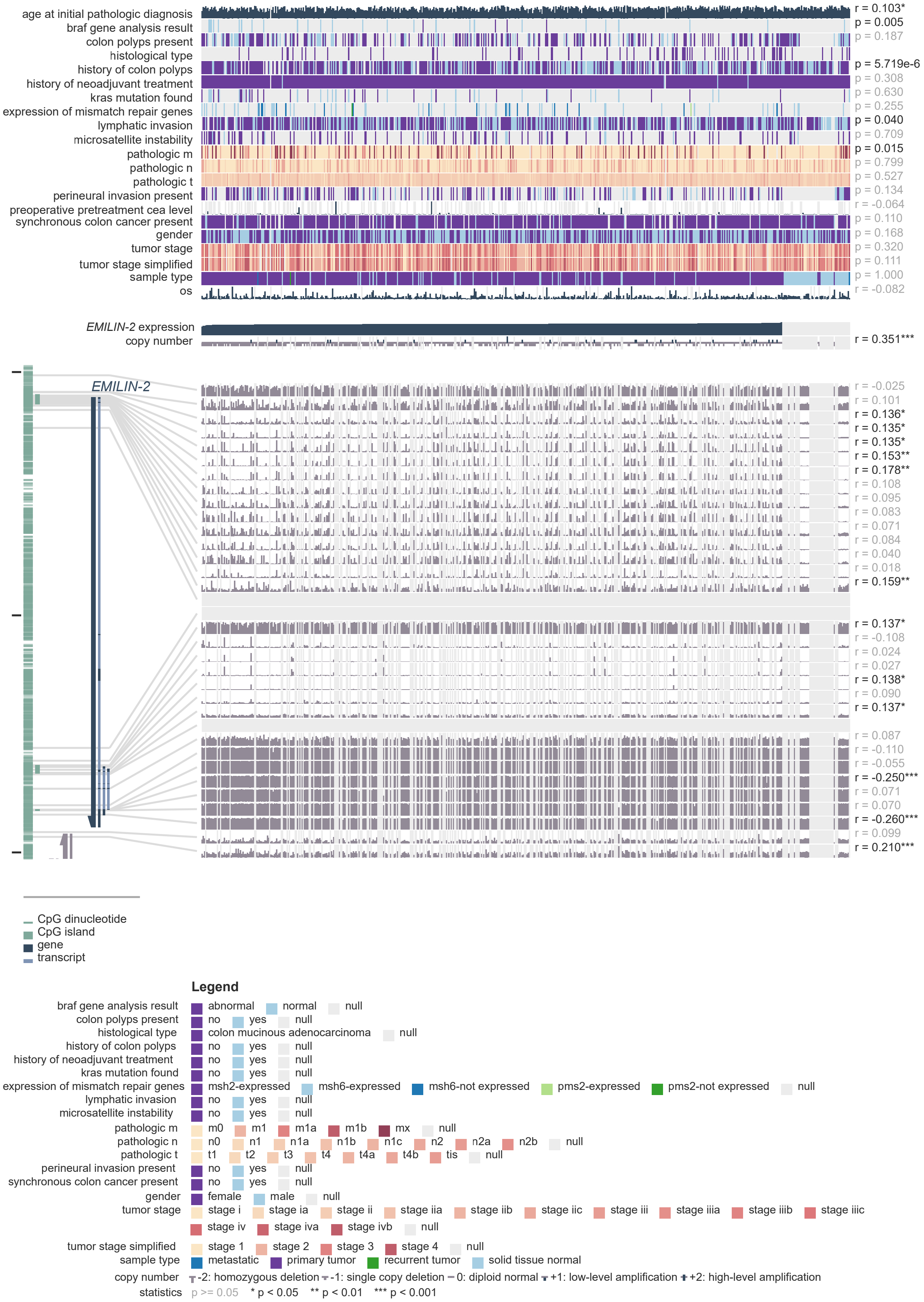
age at initial pathologic diagnosis
braf gene analysis result
colon polyps present
histological type
history of colon polyps
history of neoadjuvant treatment
kras mutation found
expression of mismatch repair genes
lymphatic invasion
microsatellite instability
pathologic m
pathologic n
pathologic t
perineural invasion present
preoperative pretreatment cea level
synchronous colon cancer present
gender
tumor stage
tumor stage simplified
sample type
os
r = 0.103*
p = 0.005
p = 0.187
p = 5.719e-6
p = 0.308
p = 0.630
p = 0.255
p = 0.040
p = 0.709
p = 0.015
p = 0.799
p = 0.527
p = 0.134
r = -0.064
p = 0.110
p = 0.168
p = 0.320
p = 0.111
p = 1.000
r = -0.082
EMILIN-2 expression
copy number
r = 0.351***
EMILIN-2
r = -0.025
r = 0.101
r = 0.136*
r = 0.135*
r = 0.135*
r = 0.153**
r = 0.178**
r = 0.108
r = 0.095
r = 0.083
r = 0.071
r = 0.084
r = 0.040
r = 0.018
r = 0.159**
r = 0.137*
r = -0.108
r = 0.024
r = 0.027
r = 0.138*
r = 0.090
r = 0.137*
r = 0.087
r = -0.110
r = -0.055
r = -0.250***
r = 0.071
r = 0.070
r = -0.260***
r = 0.099
r = 0.210***
CpG dinucleotide
CpG island
gene
transcript
Legend
braf gene analysis result: abnormal, normal, null
colon polyps present: no, yes, null
histological type: colon mucinous adenocarcinoma, null
history of colon polyps: no, yes, null
history of neoadjuvant treatment: no, yes, null
kras mutation found: no, yes, null
expression of mismatch repair genes: msh2-expressed, msh6-expressed, msh6-not expressed, pms2-expressed, pms2-not expressed, null
lymphatic invasion: no, yes, null
microsatellite instability: no, yes, null
pathologic m: m0, m1, m1a, m1b, mx, null
pathologic n: n0, n1, n1a, n1b, n1c, n2, n2a, n2b, null
pathologic t: t1, t2, t3, t4, t4a, t4b, tis, null
perineural invasion present: no, yes, null
synchronous colon cancer present: no, yes, null
gender: female, male, null
tumor stage: stage i, stage ia, stage ii, stage iia, stage iib, stage iic, stage iii, stage iiia, stage iiib, stage iiic, stage iv, stage iva, stage ivb, null
tumor stage simplified: stage 1, stage 2, stage 3, stage 4, null
sample type: metastatic, primary tumor, recurrent tumor, solid tissue normal
copy number: -2: homozygous deletion, -1: single copy deletion, 0: diploid normal, +1: low-level amplification, +2: high-level amplification
statistics: p >= 0.05, * p < 0.05, ** p < 0.01, *** p < 0.001

Figure S1. The *EMILIN-2* gene is methylated in CRC. Epigenetic landscape of the *EMILIN-2* gene and clinical and molecular parameters characterizing the COAD cohort (477 colorectal cancer patients) from the Cancer Genome Atlas (TCGA), as assessed by MEXPRESS (accessed on 12 March 2021). The analyses are in relation to the levels of *EMILIN-2*. The Pearson correlation coefficients are reported on the right and highlight a significant correlation between the expression of the *EMILIN-2* gene, the methylation probes, and the corresponding clinical parameters.

Figure S2

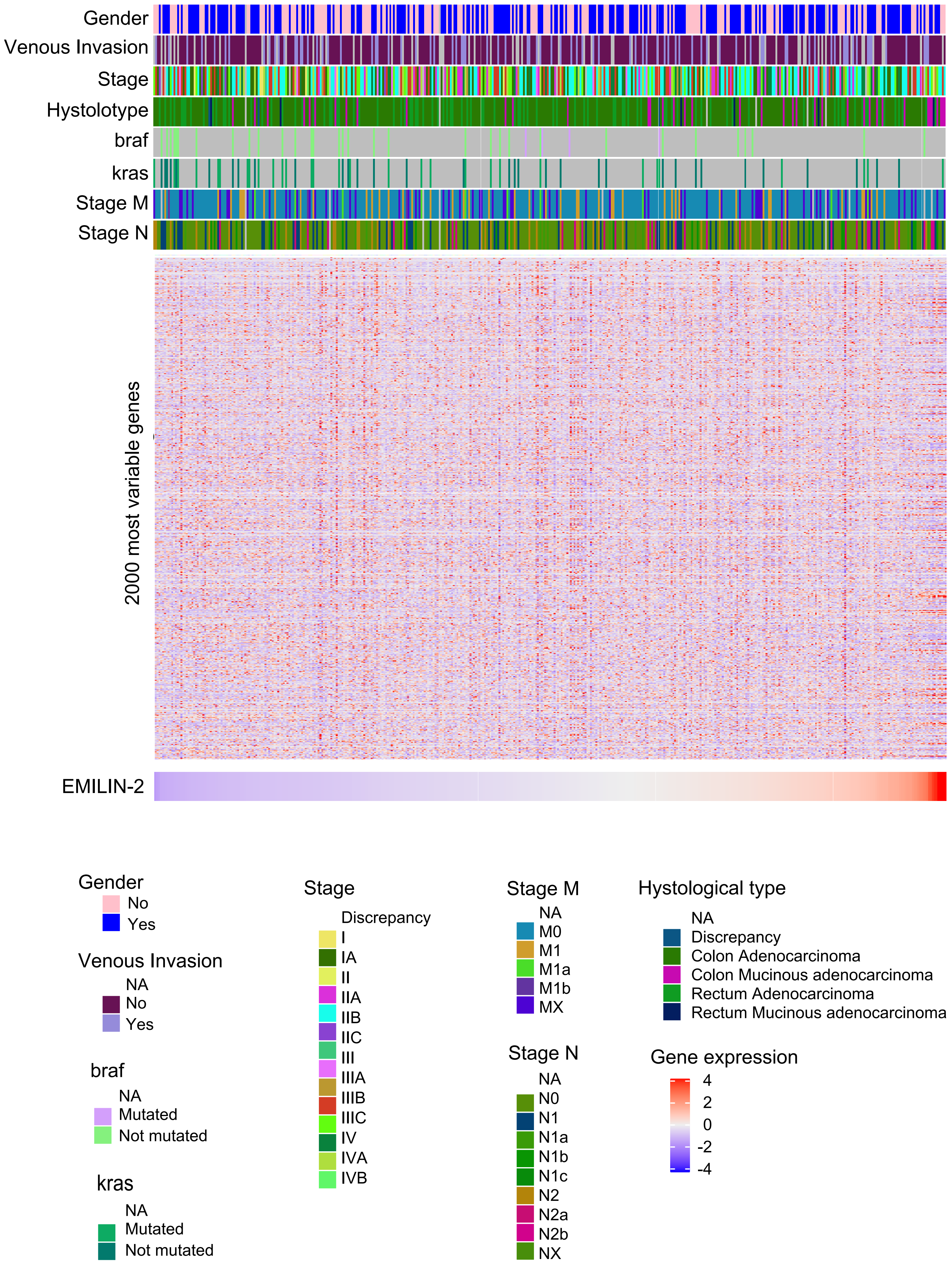
Gender
Venous Invasion
Stage
Hystolotype
braf
kras
Stage M
Stage N
2000 most variable genes
EMILIN-2
Gender
No
Yes
Venous Invasion
NA
No
Yes
braf
NA
Mutated
Not mutated
kras
NA
Mutated
Not mutated
Stage
Discrepancy
I
IA
II
IIA
IIB
IIC
III
IIIA
IIIB
IIIC
IV
IVA
IVB
Stage M
NA
M0
M1
M1a
M1b
MX
Stage N
NA
N0
N1
N1a
N1b
N1c
N2
N2a
N2b
NX
Hystological type
NA
Discrepancy
Colon Adenocarcinoma
Colon Mucinous adenocarcinoma
Rectum Adenocarcinoma
Rectum Mucinous adenocarcinoma
Gene expression
4
2
0
-2
-4

Figure S2. **The expression of EMILIN-2 is variable among CRC patients**. Stratification of the patients of the TCGA COAD cohort based on the EMILIN-2 protein levels; molecular and clinical parameters are indicated and detailed on the bottom. The heat map shows the levels of the 2000 most variable genes.

Figure S3

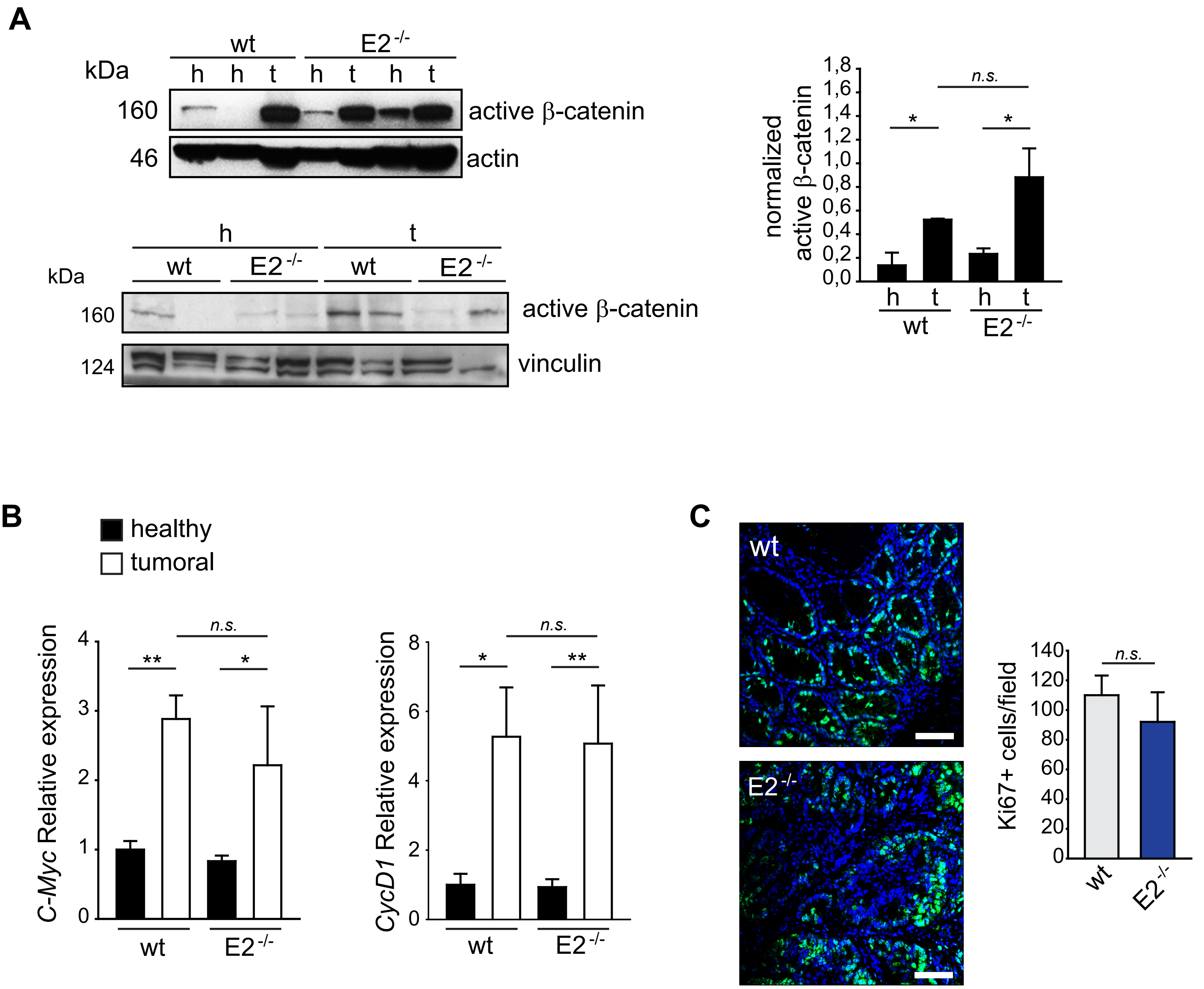

Figure S3. The higher tumor rate in *Emilin-2*$^{-/-}$ mice does not hinge on the activation of the Wnt/β-Catenin pathway. A, Representative Western blot analyses (left) and quantification (right) of β-catenin activation in the healthy (h) and tumoral (t) colonic mucosa of *wild type* (wt) and *Emilin-2*$^{-/-}$ (E2$^{-/-}$) mice; n=3. **B,** Relative expression levels of c-Myc (left) and Cyclin D1 (CycD1, right) in the healthy and tumoral colonic mucosa of *wild type* (wt) and *Emilin-2*$^{-/-}$ (E2$^{-/-}$) mice. **C**, Representative images (left) and quantification (right) of the Ki67 stainings performed on adenomas developed in *wild type* (wt) and *Emilin-2*$^{-/-}$ (E2$^{-/-}$) mice. Blue: nuclei; green: Ki67, scale bar=50 μm. Graphs represent the mean ± SD of at least three independent experiments; in A and B, *P* values were obtained using the one-way ANOVA test and in C the paired Student's t-test; *P<0.05, **P<0.01, n.s.: P>0.05.
