# Supplementary material for: Colorectal cancer development is affected by the ECM molecule EMILIN-2 hinging on macrophage polarization via the TLR-4/MyD88 pathway
Source: J Exp Clin Cancer Res. 2022 Feb 11;41:60. doi: 10.1186/s13046-022-02271-y (PMC8840294; doi:10.1186/s13046-022-02271-y)
Supplement: Supplementary file 4 — Additional file 4: Figure S4-Figure S12. [file 13046_2022_2271_MOESM4_ESM.pdf]

Figure S4

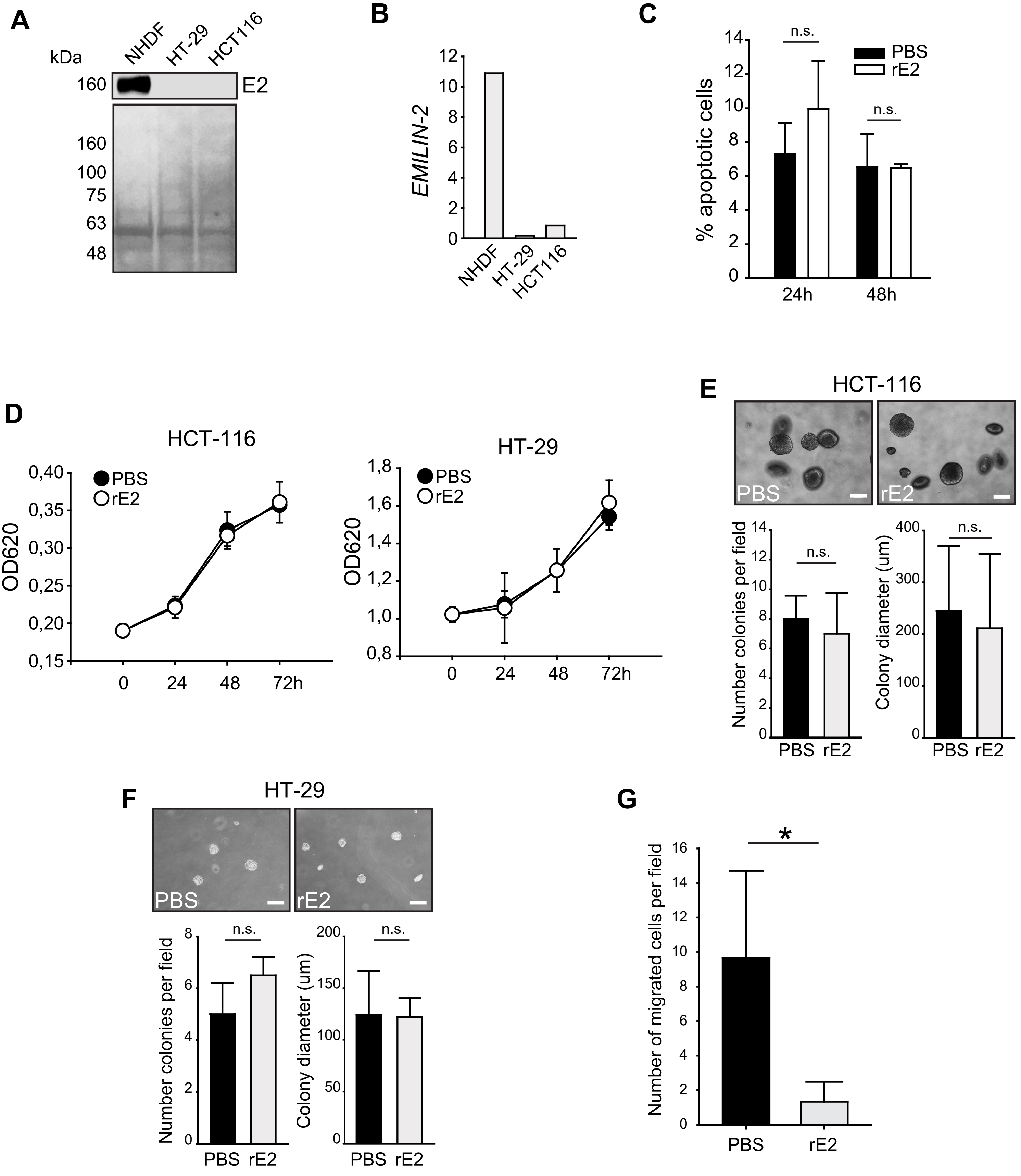
A
kDa
NHDF
HT-29
HCT116
160
E2
160
100
75
63
48
B
EMILIN-2
12
10
8
6
4
2
0
NHDF
HT-29
HCT116
C
% apoptotic cells
n.s.
n.s.
PBS
rE2
24h
48h
D
HCT-116
OD620
0,40
0,35
0,30
0,25
0,20
0,15
PBS
rE2
0
24
48
72h
HT-29
1,8
1,6
1,4
1,2
1,0
0,8
E
HCT-116
PBS
rE2
Number colonies per field
Colony diameter (um)
n.s.
n.s.
F
HT-29
PBS
rE2
Number colonies per field
Colony diameter (um)
n.s.
n.s.
G
*
Number of migrated cells per field
PBS
rE2

Figure S4. EMILIN-2 does not affect CRC cell viability. A, Western blot analysis of the EMILIN-2 (E2) expression in conditioned media (CM) from HT-29 and HCT-116 cells (top), the Ponceau-stained membrane indicates equal protein loading (bottom). CM from human fibroblasts (NHDF) was used as positive control. **B**, qPCR of the *EMILIN-2* (E2) expression, relative to that of *GAPDH*, in HT-29 and HCT-116 cells; human fibroblast (NHDF) were used as a positive control. **C**, Graph reporting the percentage of apoptotic HCT-116 cells challenged with recombinant EMILIN-2 (rE2) or vehicle (PBS), as assessed by TUNEL assay. **D**, Graph reporting the viability of HCT-116 and HT-29 cells challenged with recombinant EMILIN-2 (rE2) or vehicle (PBS), as assessed by MTT assay. **E**, Representative images (top) and quantification of the number and diameter (bottom) of the colonies formed by HCT-116 cells in 3D spheroid-based tests, following treatment with recombinant EMILIN-2 (rE2) or vehicle (PBS); scale bar=200 μm. **F**, Representative images (top) and quantification of the number and diameter (bottom) of the colonies formed by HT-29 cells in 3D spheroid-based tests, following treatment with recombinant EMILIN-2 (rE2) or vehicle (PBS); scale bar=200 μm. **G**, Graph reporting the number of migrated HCT-116 cells per field following treatment with recombinant EMILIN-2 (rE2) or vehicle (PBS), as assessed by transwell migration assays. Graphs represent the mean ± SD; *P* values were obtained with the paired Student's t-test; *$P<0.05$, n.s.: $P>0.05$.

Figure S5

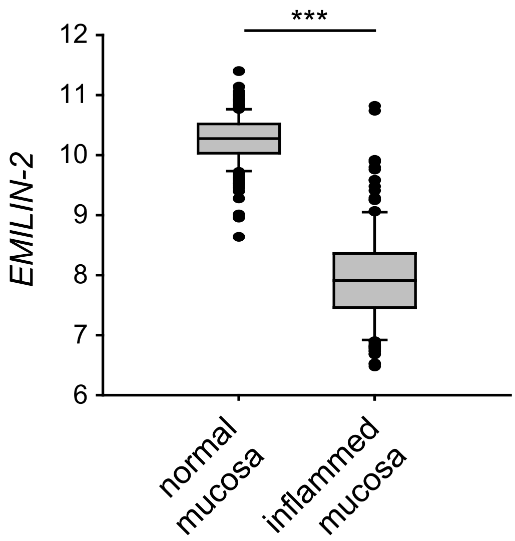

Figure S5. ***EMILIN-2* is down-regulated in patients affected by chronic inflammatory disease.** Evaluation of *EMILIN-2* gene expression in healthy and inflammed intestinal mucosa in the GEO public dataset (ID: GSE83687; healthy n=204, inflammed n=159). Graph represents the mean ± SD; the *P* value was obtained using the paired Student's t-test; ****P*<0.001.

Figure S6

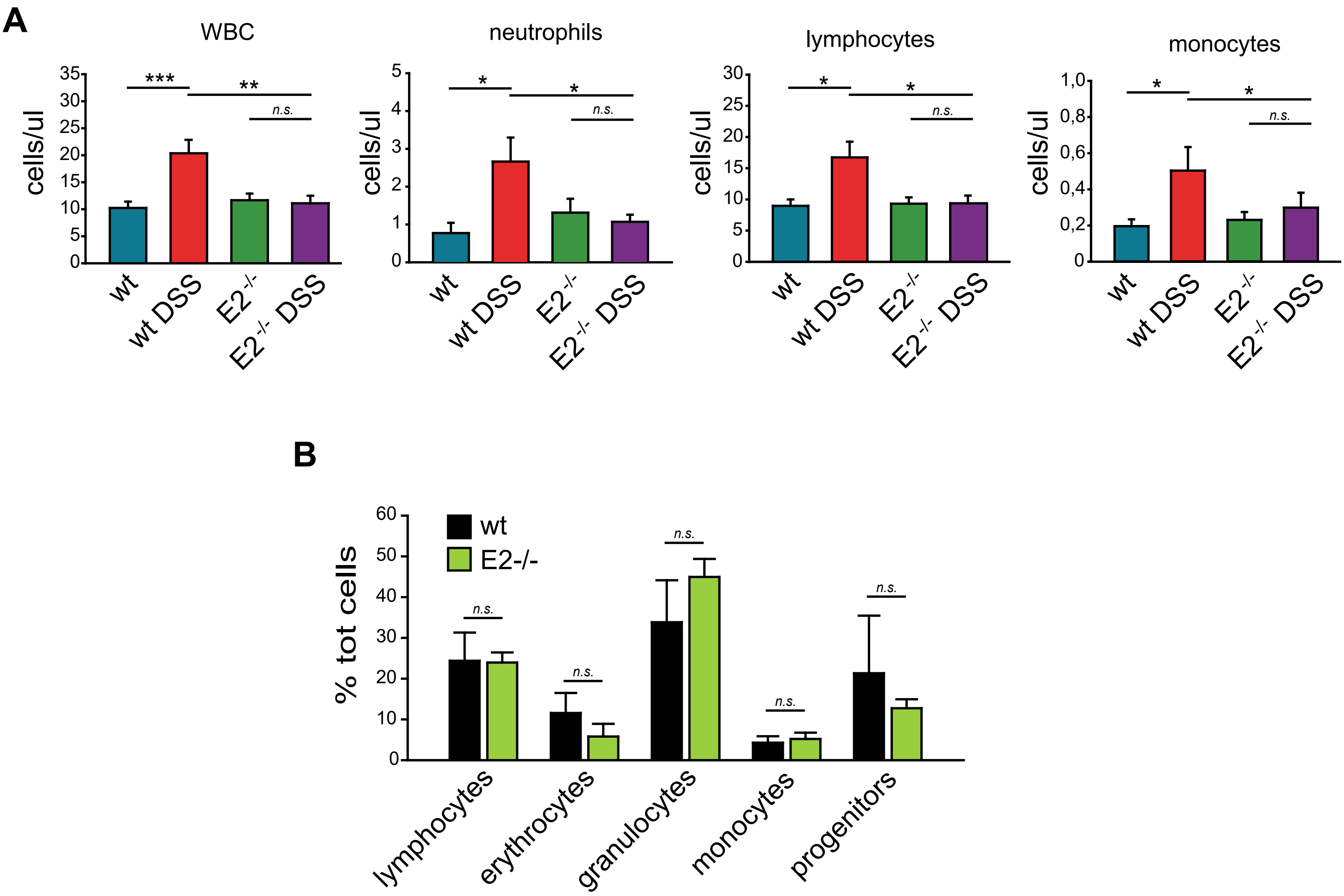

Figure S6. Ablation of EMILIN-2 impairs the number of circulating monocytes upon DSS-induced inflamation. **A,** Graphs reporting the number ($x10^3$) of circulating white blood cells (WBC), neutrophils, lymphocytes and monocytes in *wild type* (wt) and *Emilin-2*$^{-/-}$ (E2$^{-/-}$) mice treated or not with DSS for 7 days; n=8. **B,** Graphs showing the percentage of hematopoietic progenitors in *wild type* (wt) and *Emilin-2*$^{-/-}$ (E2$^{-/-}$) bone marrows, as assessed by flow cytometry; n=5. *P* values were obtained with the one-way ANOVA test. **P*<0.05, ***P*<0.01, ****P*<0.001, n.s.: *P*>0.05.

Figure S7

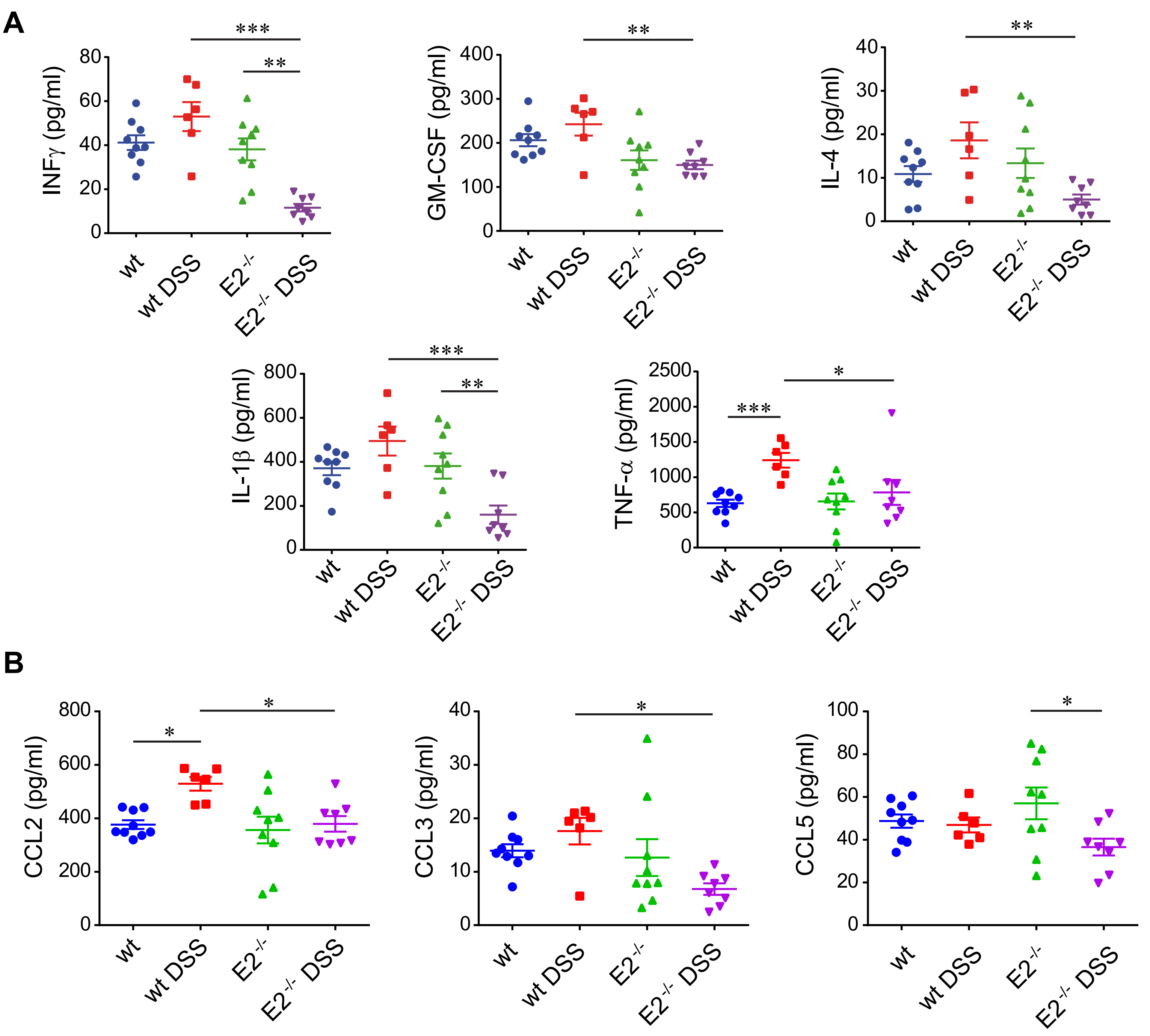

Figure S7. EMILIN-2 affects the levels of circulating inflammatory cytokynes and chemokynes during DSS-induced inflamation. A, Graphs reporting the concentration of key inflammatory cytokynes in the sera from *wild type* (wt) and *Emilin-2*$^{-/-}$ (E2$^{-/-}$) mice treated or not with DSS for 7 days. **B,** Graphs indicating the concentration of chemokynes in the sera from *wild type* (wt) and *Emilin-2*$^{-/-}$ (E2$^{-/-}$) mice treated or not with DSS for 7 days. Graphs represent the mean ± SD; *P* values were obtained with the one-way ANOVA test. * *P*<0.05, ***P*<0.01, ****P*<0.001.

Figure S8

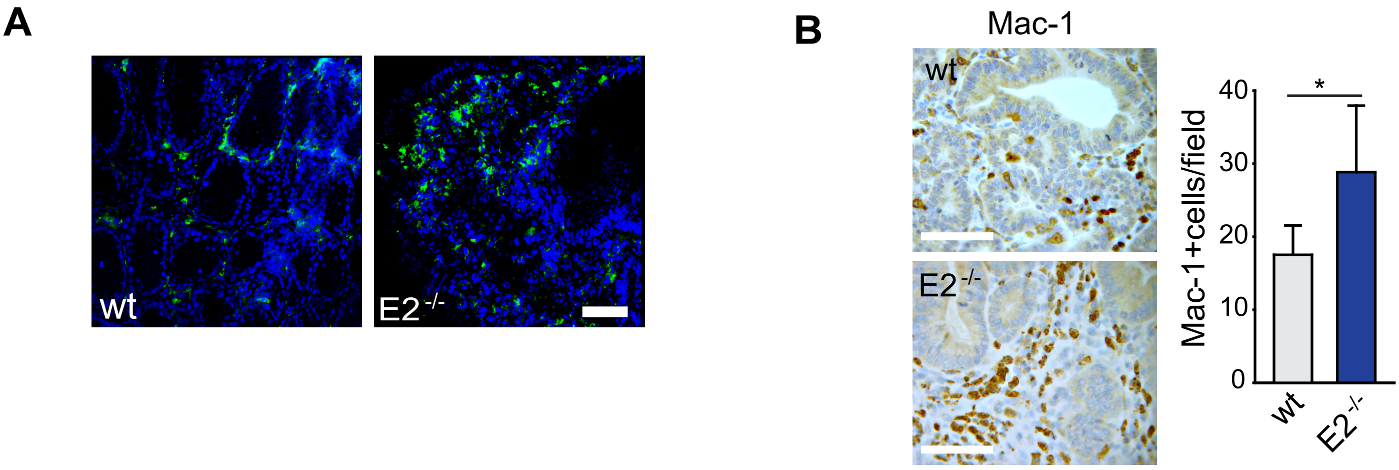

Figure S8. EMILIN-2 affects macrophage recruitment in colorectal tumors. A, Representative images of CD68 staining (green) in tumors from *wild type* (wt) and *Emilin-2*$^{-/-}$ (E2$^{-/-}$) mice treated or not with AOM/DSS; blue: nuclei, scale bar=50 µm. **B,** Representative images and quantification of Mac-1 positive cells (macrophages) in tumors from *wild type* (wt) and *Emilin-2*$^{-/-}$ (E2$^{-/-}$) mice upon AOM-induced CRC; magnification 400x; scale bar=50 µm; n=6. The graph represents the mean ± SD; *P* values were obtained using the paired Student's t-test; * P<0.05.

Figure S9

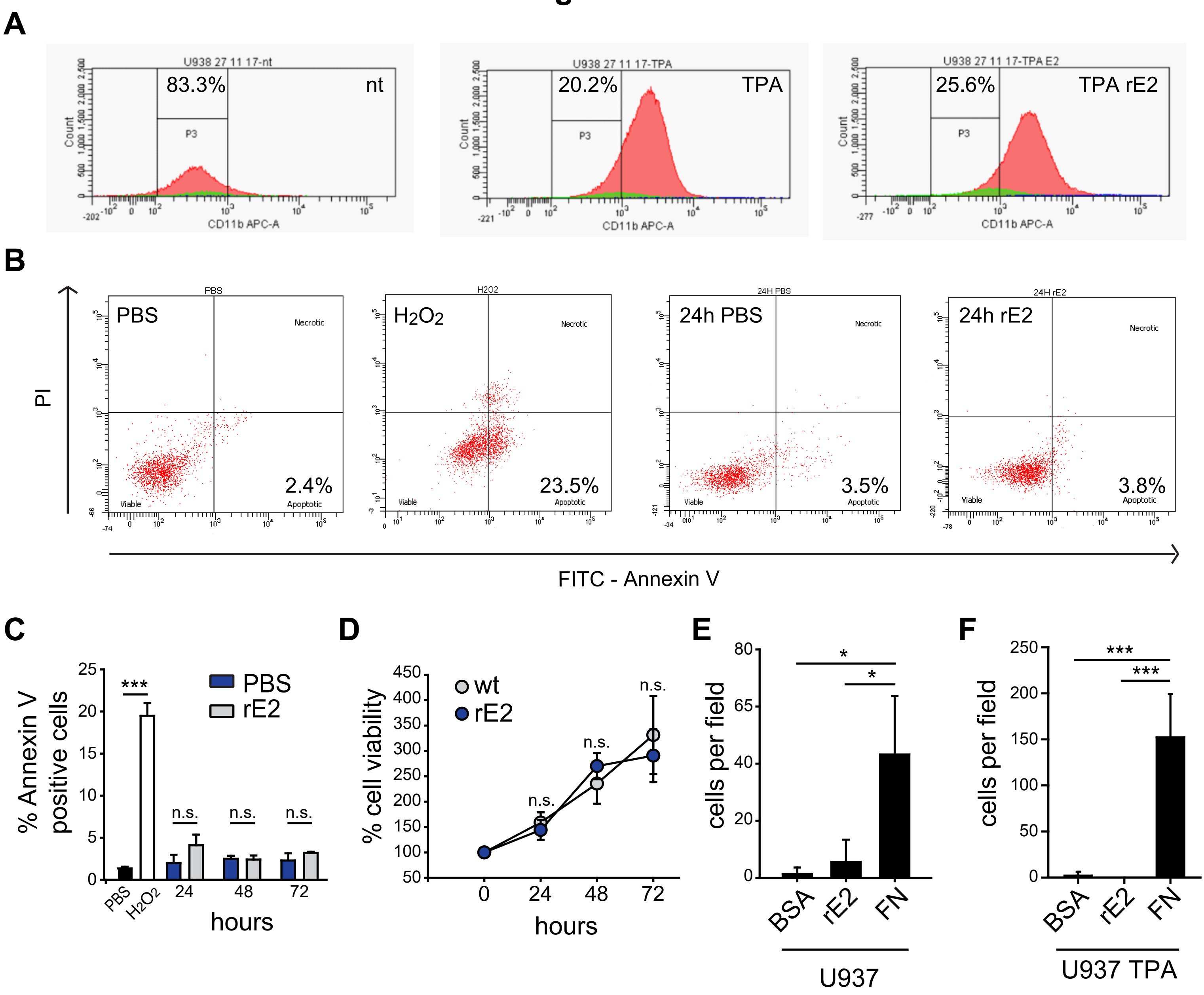

Figure S9. EMILIN-2 does not direcly impact on U937 cells activation, viability and adhesion. A, Plots showing the expression of the CD11b activation marker in the monocytic U937 cell line in the presence of TPA and/or recombinant EMILIN-2 (rE2), as evaluated by flow cytometry. **B,** Representative plots of the Annexin V assays performed on U937 cells challenged with recombinant EMILIN-2 (rE2) or vehicle (PBS). Cells treated with H_2O_2 were used as positive control. **C**, Percentage of Annexin V positive U937 cells in the presence of recombinant EMILIN-2 (rE2) or vehicle (PBS), as assessed in C. **D**, Percentage of U937 cell viability upon treatment with recombinant EMILIN-2 (rE2) or vehicle (PBS), as assessed by MTT assay. **E**, Graph reporting the number of U937 cells adhered on wells coated with EMILIN-2 (rE2), BSA or fibronectin (FN). **F**, Graph reporting the number of TPA-activated U937 cells adhered on wells coated with EMILIN-2 (rE2), BSA or fibronectin (FN). Graphs represent the mean ± SD of at least three independent experiments. In C and D, *P* values were obtained with the paired Student's t-test; in E and F, *P* values were obtained using the one-way ANOVA test; *P<0.05, ****P*<0.001, n.s.: *P*>0.05.

Figure S10

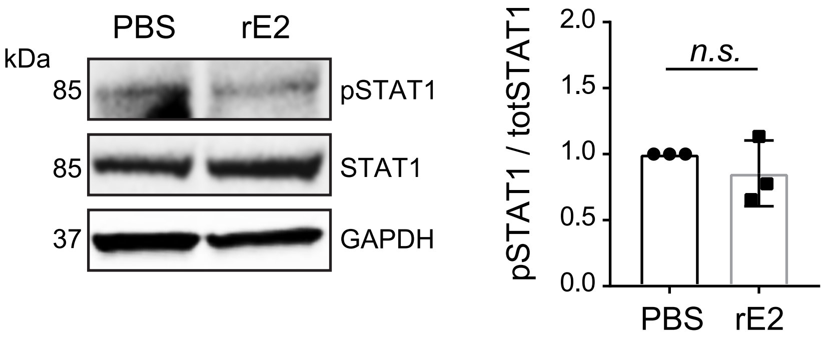

Figure S10. EMILIN-2 does not affect STAT1 phosphorilation. Representative Western blot (left) and quantification (right) of pSTAT1 in TPA-activated THP-1 cells challenged with recombinant EMILIN-2 (rE2) or vehicle (PBS); n=3. The graph represents the mean ± SD; the *P* value was obtained using the paired Student's t-test; n.s.: $P>0.05$.

Figure S11

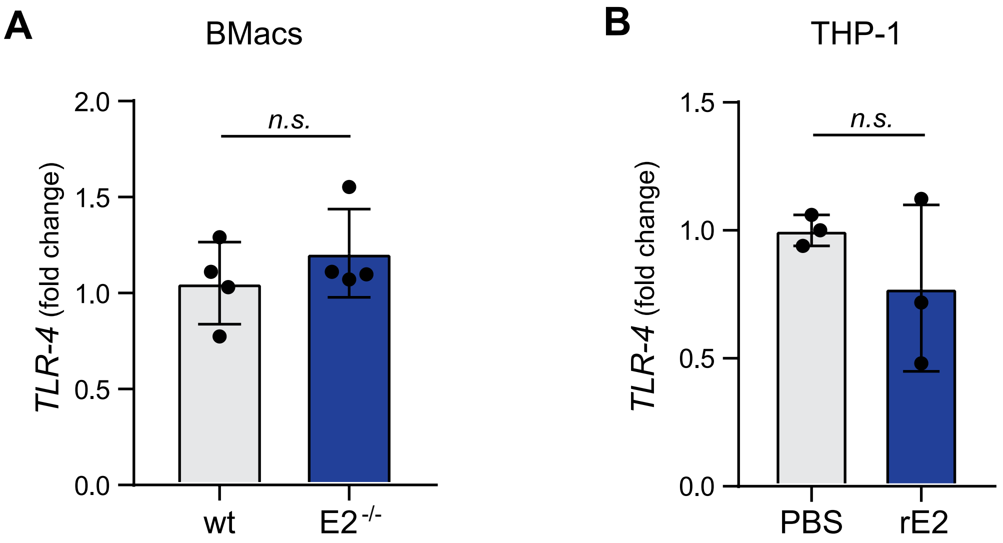

Figure S11. EMILIN-2 does not alter the expression of *TLR-4*. A, qPCR analyses of *TLR-4* expression, relative to that of *GAPDH*, in bone marrow-derived macrophages from *wild type* (wt) and *Emilin-2*$^{-/-}$ (E2$^{-/-}$) mice; n=4. **B**, qPCR analyses of *TLR-4* expression, relative to that of *GAPDH*, in TPA-activated THP-1 cells treated with recombinant EMILIN-2 (rE2) or vehicle (PBS); n=3. Graphs represent the mean ± SD of at least three independent experiments; *P* values were obtained with the paired Student's t-test; n.s.: $P>0.05$.

Figure S12

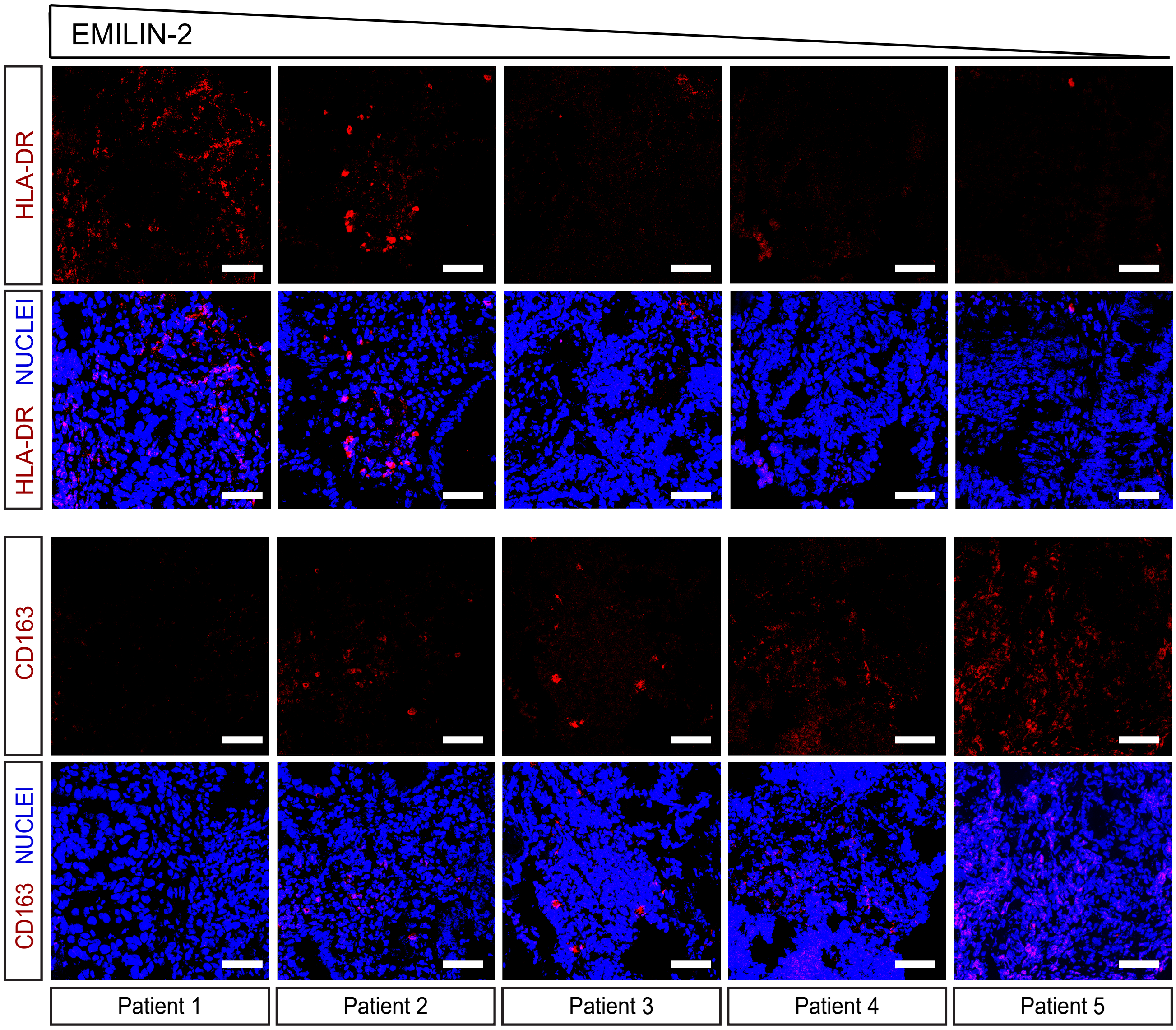

Figure S12. EMILIN-2 associates with the expression of the M1 and M2 markers in CRC patients. Representative images of immunofluorescence analyses of HLA-DR (red, top panel) and CD163 (red, low panel) in five patients displaying different EMILIN-2 expression levels (from high to low, as indicated); blue: nuclei, scale bar=50 µm.
